# ER–mitochondria contacts orchestrate mitochondrial transport via C19orf12

**DOI:** 10.1038/s41421-026-00934-0

**Published:** 2026-11-10

**Authors:** Xiayuhe Pei, Shuqian Zhang, Peiyang Li, Yutong Wu, Yiru Cheng, Xia Feng, Yiqian Wu, Xiaoshuai Huang, Ning Huang, Bei Liu, Junlin Teng, Jianguo Chen, Craig Blackstone, Pengli Zheng

**Affiliations:** 1 Key Laboratory of Cell Proliferation and Differentiation of the Ministry of Education, School of Life Sciences, Peking University, Beijing, China; 2 State Key Laboratory of Membrane Biology, School of Life Sciences, Peking University, Beijing, China; 3 Peking-Tsinghua Center for Life Sciences, Academy for Advanced Interdisciplinary Studies, Peking University, Beijing, China; 4 National Biomedical Imaging Center, College of Future Technology, Peking University, Beijing, China; 5 MassGeneral Institute for Neurodegenerative Disease, Massachusetts General Hospital, Charlestown, MA USA; 6 Department of Neurology, Massachusetts General Hospital and Harvard Medical School, Boston, MA USA; 7 State Key Laboratory of Molecular Oncology, Peking University Cancer Hospital & Institute, Beijing, China; 8 Beijing Advanced Center of Cellular Homeostasis and Aging-Related Diseases, Institute of Advanced Clinical Medicine, Peking University, Beijing, China; 9 Institute of Neuroscience, Translational Medicine Institute, Health Science Center, Xi’an Jiaotong University, Xi’an, Shaanxi China; 10 Department of Physiology and Pathophysiology, School of Basic Medical Sciences, Health Science Center, Xi’an Jiaotong University, Xi’an, Shaanxi China; 11 Center for Quantitative Biology, Academy for Advanced Interdisciplinary Studies, Peking University, Beijing, China

**Keywords:** Endoplasmic reticulum, Mitochondria, Mechanisms of disease

## Abstract

Endoplasmic reticulum (ER)–mitochondria contact sites facilitate lipid transfer, calcium signaling, and mitochondrial division; however, their role in mitochondrial transport remains unclear. Here, we identify hereditary spastic paraplegia (HSP)-associated protein C19orf12 as an ER-anchored regulator of mitochondrial trafficking. The loss of C19orf12 leads to perinuclear mitochondrial clustering in cells, impaired anterograde transport in neurons, and progressive locomotor dysfunction in mice that recapitulates key features of HSP in humans. Mechanistically, C19orf12 forms a discrete complex with the mitochondrial Rho GTPase Miro1 and the Kinesin-1 motor Kif5b, thereby providing a molecular link between ER–mitochondria contacts and microtubule-based anterograde transport. Ca^2+^ also modulated C19orf12–Miro1 behavior in vitro and showed a biphasic association with mitochondrial motility in live cells. Therefore, our findings establish ER‒mitochondria contact sites as logistical hubs for Kinesin-1 motor loading and highlight the importance of the Ca^2+^-tunable C19orf12 scaffold in sustaining directional mitochondrial transport, providing a mechanistic framework for how C19orf12 loss may contribute to neurodegeneration.

## Introduction

Cells continuously adjust the dynamic distribution of their organelles through intracellular transport to ensure the efficient and orderly execution of cellular activities. Among these organelles, mitochondria are essential metabolic hubs that participate in key cellular processes such as ATP production, calcium buffering, and apoptosis^1–3^. Mitochondrial transport is critical for cells to maintain spatial energetic and metabolic balance and is particularly indispensable for highly specialized cells with extended morphologies and intense energy demands, most notably neurons^2–5^. Neurons possess elongated axons and rely on precise mitochondrial delivery to distal synapses to sustain synaptic transmission, plasticity, and survival. Consequently, aberrant mitochondrial transport is a hallmark of numerous neurodegenerative disorders, including Alzheimer’s disease, Parkinson’s disease, and hereditary spastic paraplegia (HSP)^6–8^.

Mitochondrial trafficking is driven by a diverse repertoire of molecular motors and adaptors. Long-range anterograde transport is mediated primarily by microtubule-based Kinesin-1 (Kif5) and Kinesin-3 (Kif1b)^9^, whereas retrograde movement relies on cytoplasmic dynein^10,11^. In addition to these long-range transporters, short-range motility in actin-rich domains involves Myosin19^12,13^. These motors are coupled to the mitochondrial surface via adaptor complexes, including the Metaxins^14^ and, most notably, the mitochondrial Rho GTPases (Miro1/2)^15,16^ and trafficking Kinesin-binding proteins (TRAK1/2)^17–20^. A fundamental question thus arises: how do cells spatially coordinate these ubiquitous motors and adaptors to ensure that mitochondria are mobilized or anchored at the right time and place?

Miro1, which harbors EF-hand motifs to sense cytosolic Ca^2+^ (cCa^2+^), is a critical regulatory component of this machinery. Specifically, a sharp increase in local cCa^2+^ concentration, such as that occurring during neuronal depolarization or synaptic activity, acts as a well-recognized “stop signal” that inhibits mitochondrial movement, particularly in neurons^21,22^. While this arrest is believed to involve Ca^2+^-induced conformational changes in Miro1, the exact molecular basis for motor protein inhibition remains incompletely understood, as studies have proposed either the decoupling of Kinesin from its cargo or a direct impairment in motor protein–microtubule attachment. Additionally, this process is further modulated by interactions with the mitochondrial calcium uniporter (MCU) and mitochondrial matrix calcium dynamics^23,24^. Importantly, in contrast to these inhibitory mechanisms, the role of basal physiological Ca^2+^ levels remains less defined, although emerging evidence suggests that specific calcium concentrations may be required for the integrity of the mitochondrial transport complex rather than solely acting as a stop signal^24–27^.

As the largest membranous organelle in the cell, the endoplasmic reticulum (ER) functions as a central platform for organelle communication^28^. Through extensive membrane contact sites (MCSs), the ER orchestrates essential physiological processes, including calcium transfer^29^, lipid exchange^30^, and mitochondrial fission–fusion dynamics^31,32^. Previous work from our laboratory has demonstrated that the structural distribution of the ER broadly dictates the spatial organization of almost all other membrane-bound organelles^33^. These findings suggest that the ER may play a central, yet underappreciated role in governing broad organelle transport. In support of this idea, specific ER-resident proteins are known to regulate the transport of endosomes and lysosomes; for instance, Protrudin acts as a scaffold that simultaneously binds kinesin-1 and the endosomal protein Rab7, thereby promoting the anterograde transport of late endosomes and lysosomes^34^. Recent studies on mitochondria have further suggested that ER tubules can mechanically guide mitochondrial positioning via “hitchhiking” mechanisms^35^, and notably, the mitochondrial transport regulator Miro1 is enriched at ER–mitochondria interfaces^36^. Together, these observations suggest that ER-resident proteins may regulate mitochondrial motility; however, whether these ER-resident proteins directly recruit or modulate the mitochondrial transport machinery itself remains unknown.

HSP provides crucial information for understanding this potential link. HSP is characterized by the length-dependent degeneration of corticospinal motor neurons, leading to progressive lower limb spasticity^37^. Strikingly, more than 60% of pathogenic HSP mutations occur in ER-shaping or ER-resident proteins (e.g., Atlastins, REEPs, and Spastin^37^), indicating that ER dysfunction is a primary driver of axonopathy. The downstream consequences of these ER mutations often manifest as defects in axonal transport and mitochondrial distribution^38^. This disconnect suggests that we currently have not identified the critical molecular linkers connecting the ER network to the mitochondrial transport machinery.

*C19orf12*, a gene that is mutated in patients with a severe autosomal recessive form of HSP (spastic paraplegia type 43, SPG43) as well as a form of neurodegeneration with brain iron accumulation (NBIA), represents a candidate for this missing link^39–42^. Previous studies have reported that overexpressed C19orf12 is localized to both the ER and mitochondria and that this protein is involved in many cellular processes, such as autophagy and lipid metabolism^43,44^. However, the precise subcellular localization of the endogenous protein remains controversial, and its primary physiological function — specifically the molecular mechanism linking its dysfunction to axonal degeneration — remains elusive. Here, we identify C19orf12 as an ER-anchored organizer of the mitochondrial transport complex that is enriched at ER–mitochondria contact sites. We demonstrate that C19orf12 is indispensable for maintaining axonal integrity, as C19orf12 deficiency drives a progressive motor impairment in mice and distal mitochondrial depletion in neurons. Mechanistically, we show that C19orf12 functions as a Ca^2+^-tunable scaffold that connects the ER to the mitochondrial surface, promoting the assembly of the Miro1–Kinesin-1 complex. Our findings reveal that ER‒mitochondria contacts serve as critical logistical hubs for organelle transport, providing a unifying mechanism for the pathogenesis of HSP.

## Results

### C19orf12 deficiency leads to a progressive motor impairment and HSP-like pathology in mice

We first characterized the expression profile of a C19orf12 homolog (encoded by *1600014C10Rik*) in the mouse brain to determine the physiological function of this NBIA- and HSP-associated protein. Immunoblotting of tissue lysates from wild-type (WT) mice (*1600014C10Rik*^*+/+*^) revealed that C19orf12 expression progressively increased during postnatal development (from P5 to P60), which coincided with the period of neuronal maturation (Supplementary Fig. S1a). In contrast, *1600014C10Rik* mRNA levels did not show a corresponding significant change across the examined developmental stages (Supplementary Fig. S1b), suggesting that the developmental increase in C19orf12 protein abundance may be regulated predominantly at the post-transcriptional level.

**Fig. 1:**
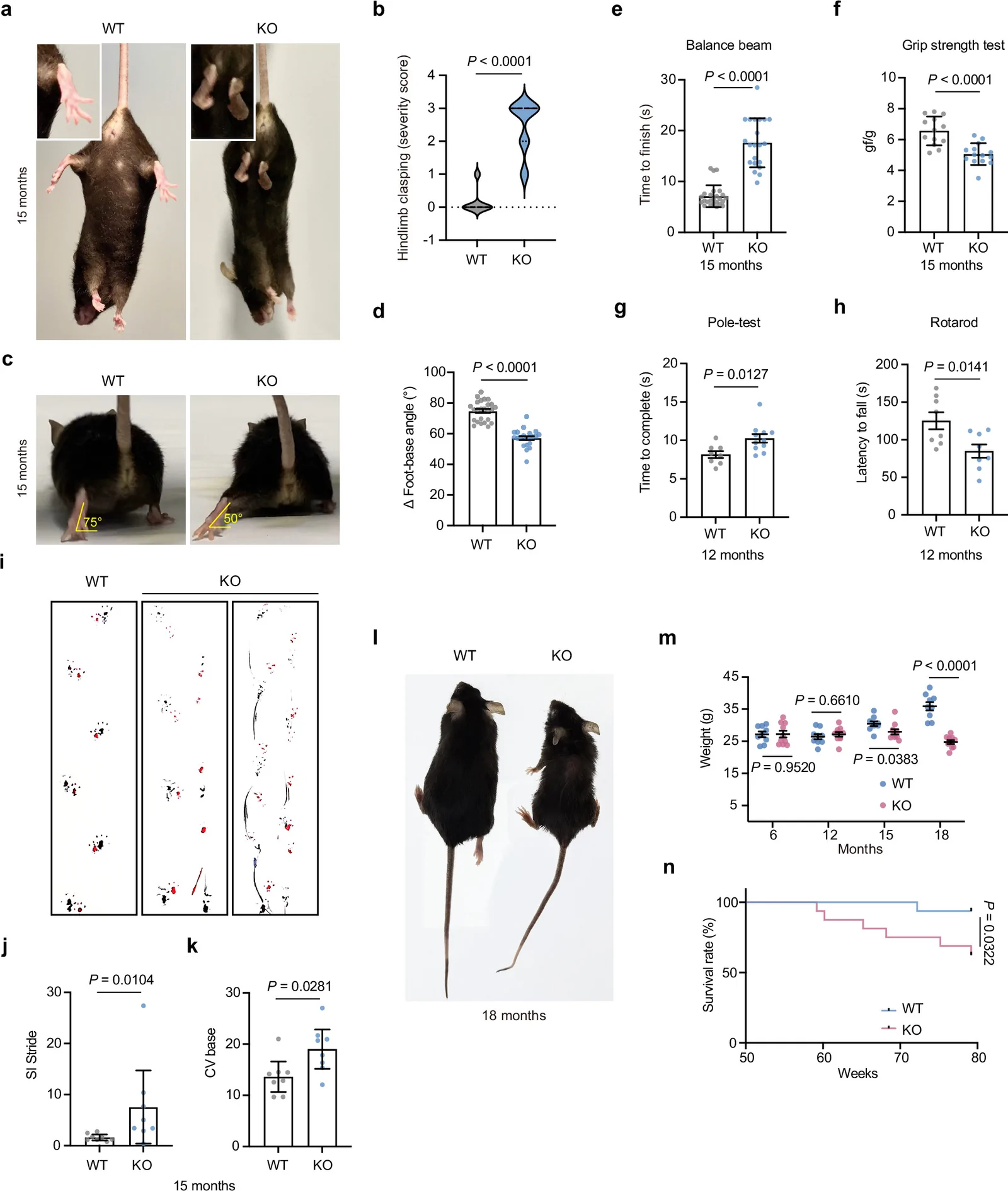
C19orf12 deficiency leads to a progressive motor impairment and HSP-like pathology in mice. **a** Representative images of 15-month-old WT mice (*1600014C10Rik*^*+/+*^) and knockout mice (*1600014C10Rik*^*–/–*^) showing the hindlimb posture during tail suspension. Insets highlight hindlimb clasping. **b** Violin plot showing hindlimb clasping scores (scale of 0–3 points) for WT (*n* = 27) and knockout (*n* = 25) mice. Each dot represents one animal. The dashed line indicates a score of 0. Statistical significance was assessed using an unpaired two-tailed *t*-test. **c** Representative images showing hindlimb splay angles during the forward locomotion of WT and knockout mice at 15 months of age. **d** Quantification of the hindlimb splay angles during locomotion shown in **c**. Each dot represents one animal (*n* = 25 WT mice and 21 knockout mice). The bars indicate the means ± SEMs. Statistical significance was assessed using an unpaired two-tailed *t*-test. **e**–**h** Motor coordination and strength were assessed by performing balance beam (**e**, *n* = 25 WT mice, 21 knockout mice), grip strength (**f**, *n* = 13 WT mice, 14 knockout mice), pole test (**g**, *n* = 8 WT mice, 11 knockout mice), and rotarod (**h**, *n* = 8 WT mice, 8 knockout mice) tests using WT and knockout mice at 12 months of age. Each dot represents one animal. The bars indicate the means ± SEMs. Statistical significance was assessed using an unpaired two-tailed *t*-test. **i**–**k** Representative gait traces (**i**) and quantification of the gait parameters (**j**, **k**), including the stride symmetry index (**j**, SI stride) and step variability (**k**, CV base), of WT and knockout mice. SI stride reflects left–right stride symmetry, with lower values indicating greater symmetry. CV base represents step-to-step variability and serves as a measure of gait stability. Each dot represents one animal (*n* = 8 mice per group). The bars indicate the means ± SEMs. Statistical significance was assessed using a two-tailed Mann–Whitney test. **l** Representative images of WT and knockout mice at 18 months of age. **m** Body weight measurements of WT and knockout mice at the indicated ages. Each dot represents one animal (*n* = 9 WT mice, 11 knockout mice). The bars indicate the means ± SEMs. Statistical significance was assessed using an unpaired two-tailed *t*-test. **n** Kaplan–Meier survival curves of WT and knockout mice (*n* = 15 WT mice, 17 knockout mice). Statistical significance was assessed using the log-rank test.

We next generated constitutive knockout mice (*1600014C10Rik*^*–/–*^) using a CRISPR/Cas9 targeting strategy (Supplementary Fig. S1c) and confirmed the complete loss of *1600014C10Rik* transcripts by RT-qPCR (Supplementary Fig. S1d). Clinically, *C19orf12* mutations cause both NBIA and SPG43, which is characterized by progressive spasticity and motor dysfunction^40^. Our mouse model robustly recapitulated these features. Notably, aged knockout mice (15 months) developed pronounced hindlimb clasping reflexes and abnormal gait patterns that were indicative of pyramidal tract dysfunction (Fig. 1a–d). Comprehensive behavioral testing revealed significant deficits in motor coordination and neuromuscular strength; compared with littermate WT controls, knockout mice showed a reduced latency on the rotarod, increased time to descend the pole, difficulty performing the beam-walking test, and weakened grip strength (Fig. 1e–h). Although the active motor assessments were terminated, we continued to monitor spontaneous behaviors and general health longitudinally. An automated gait analysis of aged mice revealed a higher stride symmetry index (SI stride) and increased coefficient of variation (CV base), quantitatively confirming persistent gait instability (Fig. 1i–k). Furthermore, longitudinal monitoring revealed a late-onset systemic phenotype characterized by progressive weight loss and increased mortality in geriatric knockout mice (Fig. 1l–n). These data establish *1600014C10Rik*^*–/–*^ mice as an in vivo model that recapitulates key motor features associated with C19orf12 deficiency and indicate that C19orf12 is essential for maintaining long-term motor function.

### C19orf12 is essential for axonal mitochondrial transport and bioenergetics in hippocampal neurons

**Fig. 2:**
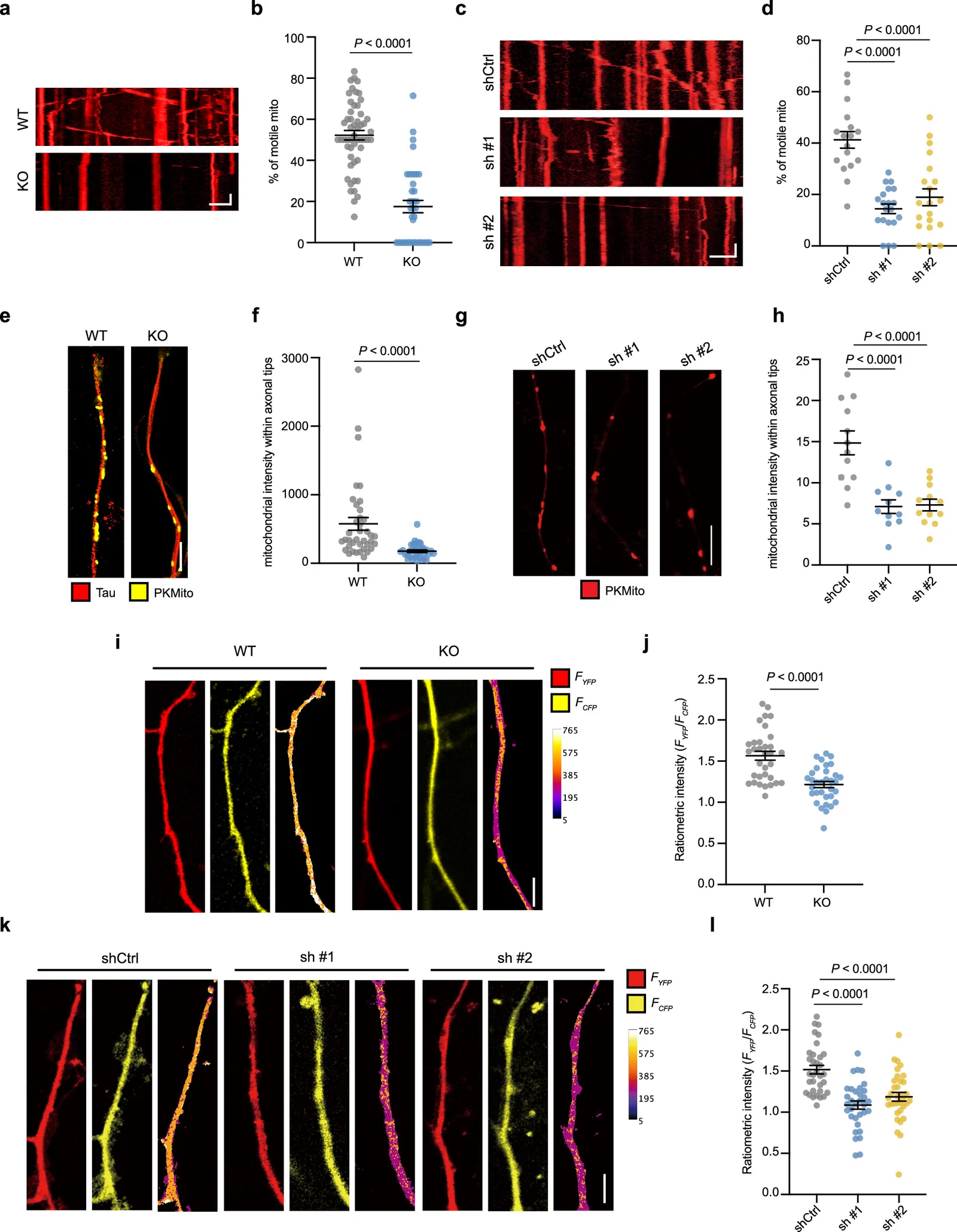
The loss of C19orf12 impairs the axonal mitochondrial distribution, motility and function. **a** Representative kymographs showing axonal mitochondrial movement in WT and knockout mouse neurons. Scale bars, 5 μm (horizontal) and 1 min (vertical). **b** Quantification of the percentage of motile mitochondria in the axons of WT (*n* = 53) and knockout (*n* = 38) mouse neurons. Each dot represents one axon. The horizontal bars indicate the means ± SEMs. Statistical significance was assessed using an unpaired two-tailed *t*-test. **c** Representative kymographs showing axonal mitochondrial movement in neurons expressing shCtrl, sh#1 or sh#2. Scale bars, 5 μm (horizontal) and 1 min (vertical). **d** Quantification of the percentage of motile mitochondria in axons under the indicated conditions in **c** (*n* = 17 shCtrl, 20 sh#1 and 20 sh#2). Each dot represents one axon. The horizontal bars indicate the means ± SEMs. Statistical significance was assessed using one-way ANOVA with multiple comparisons. **e** Representative images of axonal mitochondria labeled with PKMito in neurons derived from WT and knockout mice. Tau staining indicates axons. Scale bar, 5 μm. **f** Quantification of the mitochondrial intensity within axonal tips in WT (*n* = 38) and knockout (*n* = 34) mouse neurons. Each dot represents one axon. The horizontal bars indicate the means ± SEMs. Statistical significance was assessed using an unpaired two-tailed *t*-test. **g** Representative images of axonal mitochondria labeled with PKMito (red) in neurons expressing shCtrl, sh#1 or sh#2. Scale bar, 5 μm. **h** Quantification of the mitochondrial intensity within axonal tips under the conditions shown in **g** (*n* = 12 shCtrl, 11 sh#1 and 12 sh#2). Each dot represents one axon. The horizontal bars indicate the means ± SEMs. Statistical significance was assessed using one-way ANOVA with multiple comparisons. **i** Representative images of axonal mitochondria expressing the ratiometric mitochondrial ATP sensor ATeam1.03, shown as *F*_*YFP*_ (red), *F*_*CFP*_ (yellow), and the pseudocolor ratio (*F*_*YFP*_/*F*_*CFP*_) in the images, in WT and knockout mouse neurons. Scale bar, 5 μm. **j** Quantification of the ratiometric mitochondrial ATP signal (*F*_*YFP*_/*F*_*CFP*_) detected using ATeam1.03 in axons under the indicated conditions in **i** (*n* = 33 per group). Each dot represents one axon. The horizontal bars indicate the means ± SEMs. Statistical significance was assessed using an unpaired two-tailed *t*-test. **k** Representative images of axonal mitochondria expressing the ratiometric mitochondrial ATP sensor ATeam1.03, shown as *F*_*YFP*_ (red), *F*_*CFP*_ (yellow), and the pseudocolor ratio (*F*_*YFP*_/*F*_*CFP*_) in images, in neurons expressing shCtrl, sh#1 or sh#2. Scale bar, 5 μm. **l** Quantification of the ratiometric mitochondrial ATP signal (*F*_*YFP*_/*F*_*CFP*_) detected using the mitochondrial ATP sensor ATeam1.03 in the axons of neurons expressing shCtrl (*n* = 33), sh#1 (*n* = 33) or sh#2 (*n* = 34). Each dot represents one axon. The horizontal bars indicate the means ± SEMs. Statistical significance was assessed using one-way ANOVA with multiple comparisons.

We next investigated the cellular basis of these motor deficits. HSP is characterized by the specific degeneration of long corticospinal axons, a “dying-back” pathology that is highly consistent with defects in long-range axonal transport^37,38^, and NBIA is characterized by iron accumulation in the brain and prominent axonal spheroids, which is consistent with impaired axonal transport^41,42^. Given the crucial role of mitochondria in maintaining synaptic energy homeostasis^4^, we hypothesized that a defect in axonal mitochondrial transport is present in these mice. In primary hippocampal neurons isolated from knockout mice, time-lapse imaging revealed a severe impairment of mitochondrial motility (Fig. 2a). While WT neurons displayed robust bidirectional movement, knockout neurons exhibited a marked reduction in the fraction of motile mitochondria and a corresponding increase in the fraction of stationary mitochondria (Fig. 2a, b). This phenotype was further validated in *1600014C10Rik*-knockdown neurons using shRNA (Supplementary Fig. S1e), which recapitulated the transport defects (Fig. 2c, d). A direct consequence of failed anterograde transport is the inability to replenish mitochondria at distal sites. Imaging of axonal terminals revealed a progressive depletion of mitochondria toward the distal end in knockout neurons compared with controls (Fig. 2e). Quantification of the mitochondrial fluorescence intensity specifically within the axonal tips confirmed a significant reduction in the mitochondrial payload (Fig. 2f–h).

Mitochondria are essential for local ATP synthesis to fuel synaptic transmission. We therefore asked whether this spatial depletion compromised neuronal bioenergetics. Using the ratiometric ATP probe ATeam1.03 (single-excitation, dual-emission readout^45^), we detected lower ATP levels (*F*_*YFP*_/*F*_*CFP*_ ratio) in distal axons in both knockout and knockdown neurons than in control neurons (Fig. 2i–l). Collectively, these data demonstrate that C19orf12 is indispensable for the delivery of mitochondria to distal axons, thereby supplying the local ATP required to maintain neuronal energy homeostasis.

### C19orf12 deficiency induces perinuclear mitochondrial clustering

**Fig. 3:**
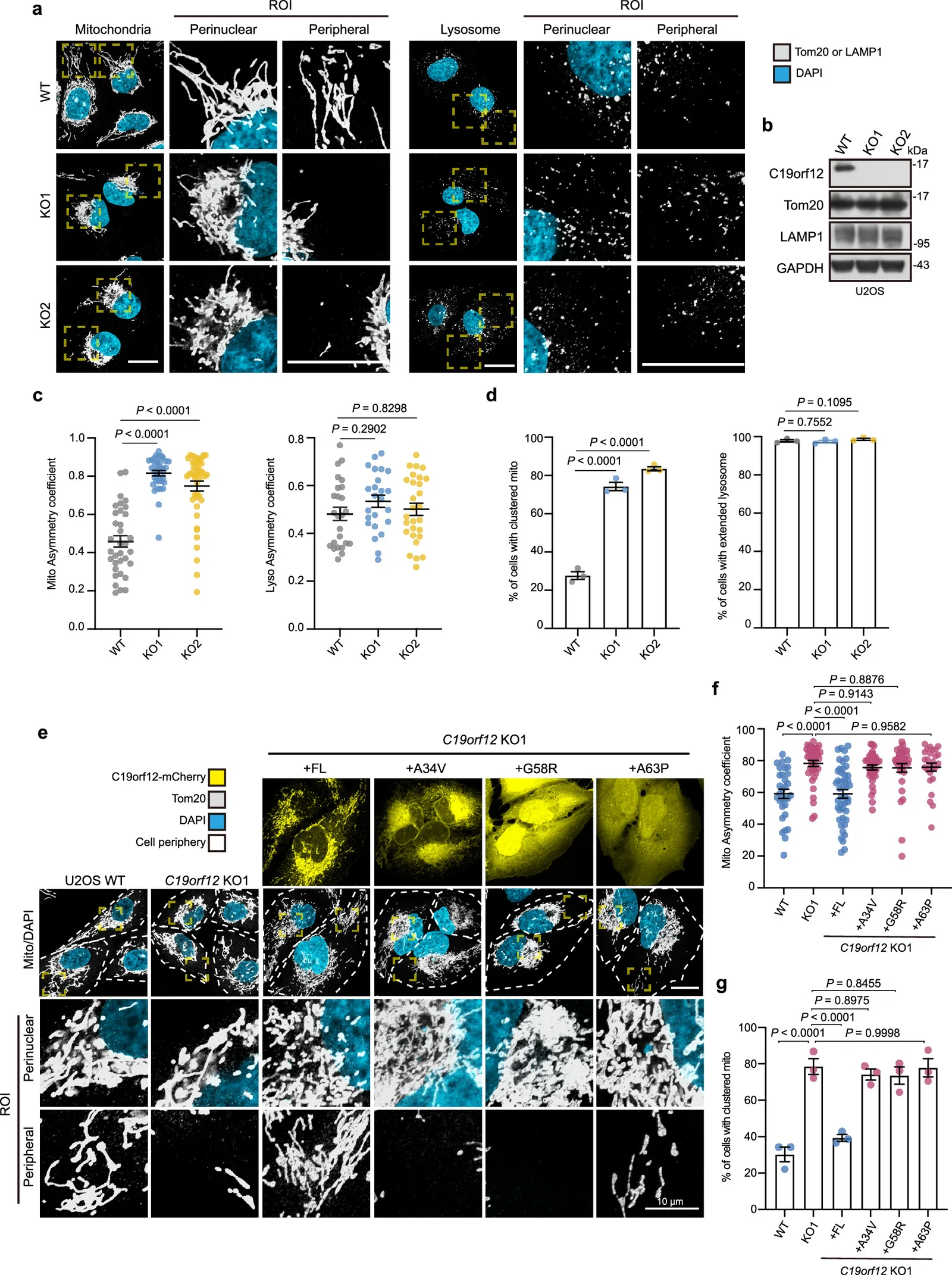
The loss of C19orf12 induces the perinuclear clustering of mitochondria in U2OS cells. **a** Representative confocal images of U2OS WT cells and two independent *C19orf12*-knockout clones stained for mitochondria (Tom20, gray) and lysosomes (LAMP1, gray). Nuclei were labeled with DAPI (blue). Boxed regions indicate ROIs. Scale bars, 20 μm. **b** Immunoblot confirming the loss of C19orf12 in two independent knockout clones. Tom20 and LAMP1 were used as mitochondrial and lysosomal markers, respectively; GAPDH served as a loading control. **c** Quantification of mitochondrial (left panel, *n* = 34, 37, and 46 from left to right) and lysosomal (right panel, *n* = 25, 24, and 28 from left to right) asymmetry coefficients in WT and *C19orf12*-knockout U2OS cells. **d** Quantification of the percentage of U2OS cells exhibiting perinuclear clustering of mitochondria (left panel) or lysosomes (right panel) following *C19orf12* knockout. *n* = 3 experiments, with at least 150 cells counted per condition. **e** Representative confocal images of the mitochondrial distribution in *C19orf12*-knockout cells re-expressing mCherry-tagged WT or mutant C19orf12 (A34V, G58R and A63P). The mitochondria were labeled with Tom20 (gray), and the nuclei were labeled with DAPI (blue). The cell periphery is outlined by white dashed lines. Boxed regions indicate ROIs. Scale bars, 20 μm (main images) and 10 μm (magnified ROIs). **f**, **g** Quantification of the mitochondrial asymmetry coefficient (**f**, *n* = 31, 39, 45, 36, 33, and 26 from left to right) and percentage of cells with perinuclear mitochondrial clustering (**g**, *n* = 3 experiments, with at least 150 cells counted per condition) under the indicated conditions in **e**. All quantitative data are shown as the means ± SEMs. In **c** and **f**, each dot represents one cell, and statistical significance was assessed using one-way ANOVA with multiple comparisons. In **d** and **g**, each dot represents one biological replicate, and the statistical significance was determined using the Kruskal‒Wallis test with multiple comparisons.

We used U2OS cells as a tractable model to elucidate the molecular mechanism underlying this transport defect, since C19orf12 is broadly expressed in various tissues^46^ (Supplementary Fig. S2a). Consistent with the neuronal phenotype, the loss of C19orf12 resulted in a striking change in the mitochondrial distribution: mitochondria were depleted from the cell periphery and substantially accumulated in the perinuclear region (Fig. 3a, b). A quantitative analysis using the asymmetry coefficient confirmed a significant clustering of mitochondria (Fig. 3c), and the proportion of cells exhibiting severe clustering increased from ~25% in WT cells to ~80% in U2OS knockout cells (Fig. 3d). Crucially, this positioning defect was specific to mitochondria. The distribution of lysosomes and protein level of a lysosomal protein LAMP1 remained unaffected (Fig. 3a, c, d; Supplementary Fig. S2b), ruling out a generalized impairment of organelle trafficking. Moreover, the mitochondrial membrane potential (Δ*ψm*) was fully preserved in the knockout cells (Supplementary Fig. S2c, d). Although modest reductions in ATP and ROS levels were observed in one knockout clone, these changes were not reproduced across independent clones (Supplementary Fig. S2e, f). The abundance of the ER stress marker GRP78/BiP was also unchanged in both knockout clones (Supplementary Fig. S2g). Collectively, these measurements do not support a reproducible global bioenergetic, oxidative, or ER stress response to C19orf12 loss under basal conditions. Finally, we confirmed that this phenotype is a conserved mechanism, as identical mitochondrial clustering was observed across multiple cell lines, including COS7 and A549 cells (Supplementary Fig. S3).

We performed rescue experiments with human *C19orf12* variants to link this phenotype to HSP pathology^39,41^. Re-expression of full-length C19orf12 in knockout cells fully restored the peripheral mitochondrial network, whereas the expression of the HSP pathogenic mutants G58R and A63P, which were diffusely distributed in the cytosol, failed to rescue the phenotype (Fig. 3e–g; Supplementary Fig. S2h). Notably, the A34V mutant retained ER localization similar to that of the WT protein but still failed to reinstate a normal distribution (Fig. 3e). This result implies that while membrane targeting is necessary, it is insufficient for function; the A34V mutation likely disrupts a specific effector interaction required for mitochondrial positioning.

We determined whether these transport defects in mice and cells were secondary to the degradation of the transport machinery by analyzing protein abundance. Immunoblotting of whole-brain and spinal cord lysates from knockout mice, as well as lysates from *C19orf12*-knockout cells, revealed that the total levels of key transport components (such as Miro1, Kif5b, and KLCs) and mitochondrial markers (Tom20) remained unchanged (Supplementary Fig. S4). These findings indicate that the observed phenotypes arise from the functional impairment of the transport machinery rather than the loss of its components.

### C19orf12 is a dynamic ER-anchored protein enriched at ER–mitochondria contact sites

**Fig. 4:**
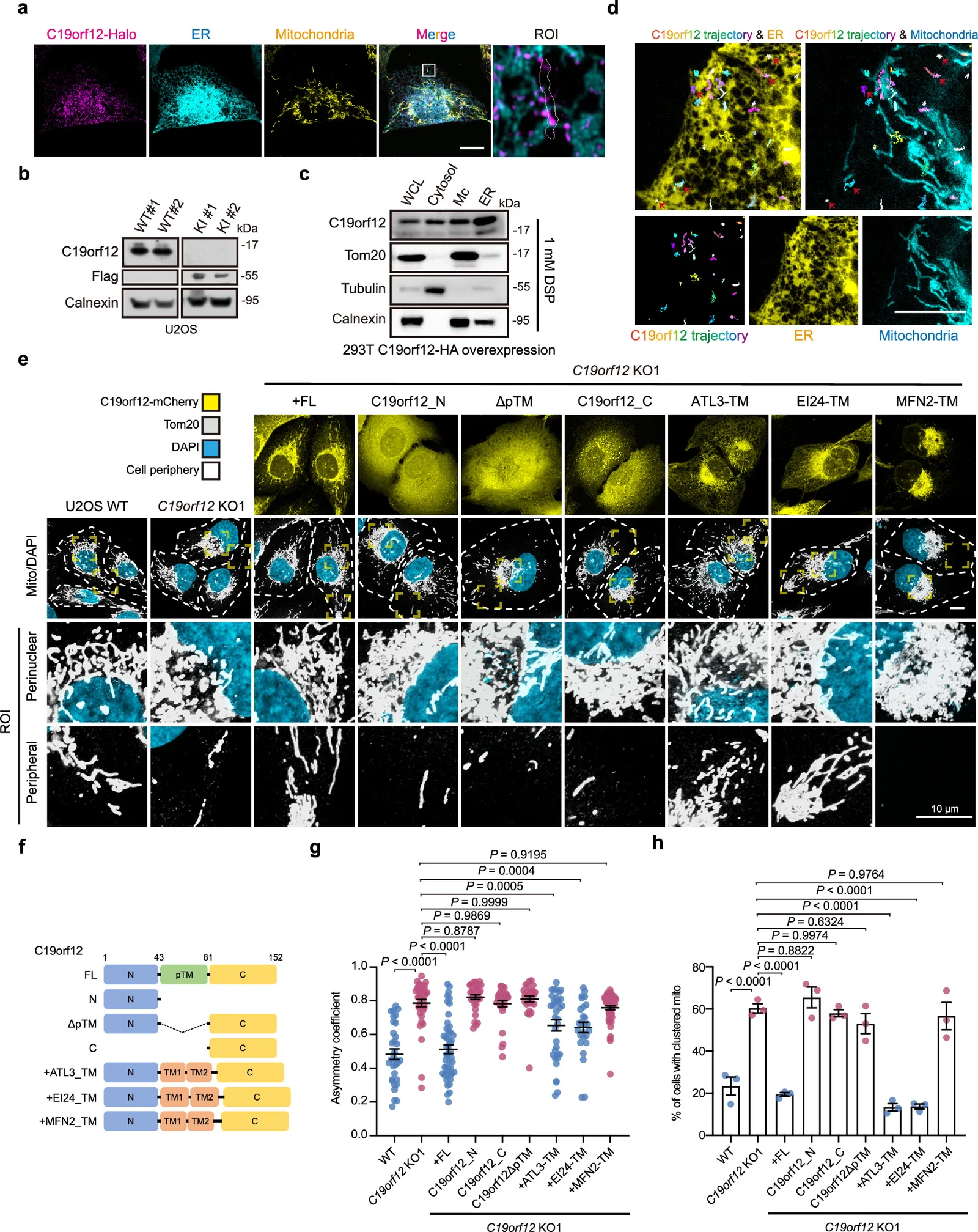
C19orf12 is a dynamic ER-anchored protein that is enriched at ER–mitochondria contact sites, and its ER localization is essential for regulating the mitochondrial distribution. **a** Representative confocal images of U2OS C19orf12–Halo knock-in cells transiently expressing the ER marker Sec61β (cyan) and stained for mitochondria (Tom20, yellow). The merged image shows C19orf12 localization relative to the ER and mitochondria. Boxed regions indicate ROIs shown at higher magnification. Scale bar, 20 μm. **b** Immunoblot-based confirmation of the *C19orf12*-Halo knock-in allele in U2OS clones. **c** Subcellular fractionation of U2OS cells after chemical cross-linking followed by an immunoblot analysis of whole-cell lysate (WCL), cytosol, crude mitochondria (Mc) and ER-enriched (ER) fractions. Tom20, Tubulin and Calnexin were used as mitochondrial, cytosolic and ER markers, respectively. **d** Single-molecule tracking of C19orf12 in U2OS *C19orf12*–Halo knock-in cells transiently expressing Sec61β (yellow) and labeled with the mitochondrial dye PKMito (cyan). Representative trajectories illustrate the dynamic movement of C19orf12 relative to the ER and mitochondria. Red arrows indicate C19orf12 molecules moving along the ER network that do not overlap with mitochondria. Scale bar, 20 μm. **e** Representative confocal images of the mitochondrial distribution in *C19orf12*-knockout cells re-expressing mCherry-tagged WT or the indicated truncation and transmembrane-domain chimeric constructs. The mitochondria were labeled with Tom20 (gray), and the nuclei were labeled with DAPI (blue). Tubulin staining is shown as a reference for cellular morphology. Scale bars, 20 μm (main images) and 10 μm (magnified ROIs). **f** Schematic representation of the C19orf12 domain deletion and chimeric constructs used for the rescue experiments in **e**. The native predicted transmembrane (pTM) domain of C19orf12 was deleted (ΔpTM) or replaced with ER-targeting TMs (from ATL3 or EI24) or a mitochondria-targeting TM (from MFN2). **g** Quantification of mitochondrial asymmetry coefficients under the indicated conditions in **e** (*n* = 32, 40, 49, 32, 31, 29, 35, 27, and 53 from left to right). Each dot represents one cell; the data are shown as the means ± SEMs. Statistical significance was assessed using one-way ANOVA with multiple comparisons. **h** Quantification of the percentage of cells exhibiting perinuclear clustering of mitochondria under the indicated conditions in **e** (*n* = 3 experiments, with at least 150 cells counted per condition). Each dot represents one biological replicate, and the statistical significance was determined using the Kruskal‒Wallis test with multiple comparisons.

Having established that C19orf12 deficiency causes mitochondrial clustering, we next sought to define its precise subcellular localization and dynamics to understand how C19orf12 exerts this spatial control. Consistent with previous reports^40^, overexpressed C19orf12 showed both ER and mitochondrial localization (Supplementary Fig. S5a). We established C19orf12-Halo-3×Flag and C19orf12-ALFA knock-in U2OS cells using CRISPR/Cas9 technology to confirm this result using the endogenous protein without artifacts due to overexpression (Fig. 4a, b; Supplementary Fig. S5b). All signals for the endogenous C19orf12 protein co-localize with the ER marker Sec61β, with a slight enrichment at ER–mitochondria contact sites (Fig. 4a; Supplementary Fig. S5b), suggesting that C19orf12 is an ER-associated protein that may specifically function at the interface between these two organelles.

However, standard subcellular fractionation^47^ yielded a paradoxical result: while the protein appeared to be associated with the ER in images, the biochemical analysis of U2OS and HEK293T cell lysates showed that the vast majority of C19orf12 was present in the cytosolic fraction, with minimal signal detected in the crude mitochondrial (Mc) or ER-enriched fractions (Supplementary Fig. S5c, d). This discrepancy suggested that C19orf12 might be loosely or dynamically associated with the ER membrane, making it susceptible to dissociation during cell lysis. We tested this hypothesis by cross-linking the proteins and subsequently performing fractionation to stabilize transient interactions. Strikingly, under cross-linking conditions, the endogenous C19orf12 pool shifted from the cytosolic fraction to the ER and mitochondrion-bound fractions (Fig. 4c). Single-molecule tracking (SMT) further confirmed that C19orf12 molecules diffuse along ER tubules but exhibit transient confinement specifically in regions overlapping with mitochondria (Fig. 4d; Supplementary Videos S1 and S2). Collectively, these biochemical and biophysical data indicate that C19orf12 is a dynamic ER-anchored protein that is transiently enriched at ER–mitochondria contact sites.

### ER membrane anchoring is necessary for C19orf12 function

We performed rescue experiments with chimeric constructs to test whether ER anchoring is required for C19orf12 function. A mutant lacking the previously predicted hydrophobic transmembrane-like region (ΔpTM) failed to rescue mitochondrial clustering (Fig. 4e–h). Our earlier biochemical data (Fig. 4c; Supplementary Fig. S5c, d) established that C19orf12 is not an integral membrane protein and that this predicted hydrophobic region does not function as a canonical transmembrane domain. However, the loss-of-function phenotype of the ΔpTM mutant suggests that this hydrophobic region is critical for the dynamic association of C19orf12 with the ER membrane (Fig. 4e). Collectively, these results indicate that ER membrane association is essential for the ability of C19orf12 to regulate mitochondrial distribution.

We next tested whether replacing the native hydrophobic region of C19orf12 with heterologous ER-targeting sequences could substitute for the endogenous motif to further confirm that ER anchoring is indispensable for the function of this protein. We generated chimeric C19orf12 constructs by replacing the predicted hydrophobic region with ER-targeting transmembrane domains from Atlastin-3 (ATL3-TM) or EI24 (EI24-TM) while also generating a control construct targeting C19orf12 to the mitochondrial outer membrane using the MFN2 transmembrane domain (MFN2-TM). Remarkably, the ER-anchored chimeric constructs (ATL3-TM and EI24-TM) fully rescued the mitochondrial distribution defects in *C19orf12*-knockout cells. In contrast, the mitochondrion-targeted MFN2-TM chimeric construct failed to restore the normal mitochondrial distribution (Fig. 4e–h; Supplementary Fig. S5e). Thus, we conclude that C19orf12 function requires an interaction with the ER membrane.

### C19orf12 is required for Kinesin-1 recruitment and interacts with the mitochondrial transport machinery

**Fig. 5:**
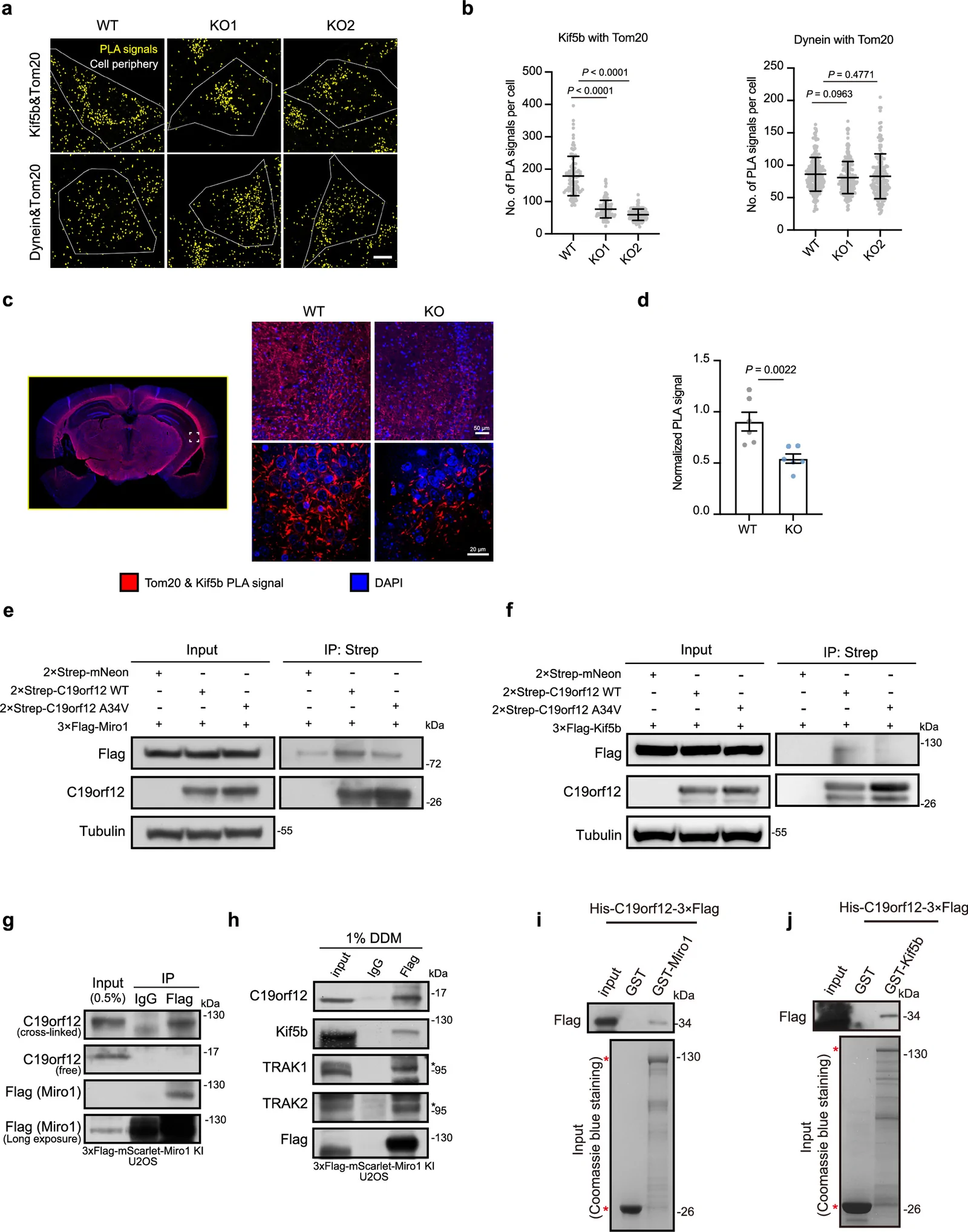
C19orf12 scaffolds the assembly of the Miro1–Kinesin anterograde transport complex. **a** Representative images of the proximity ligation assay (PLA) showing interactions between Kif5b and mitochondria (Kif5b–Tom20, top panel) or between dynein and mitochondria (Dynein–Tom20, bottom panel) in U2OS WT and *C19orf12*-knockout cells. PLA signals are shown in yellow; the cell periphery is outlined. Scale bar, 10 μm. **b** Quantification of the number of PLA signals per cell for Kif5b–Tom20 (left panel, *n* = 99, 113, 112 from left to right) and Dynein–Tom20 (right panel, *n* = 217, 246, 168 from left to right) interactions under the indicated conditions. Each dot represents one cell; horizontal bars indicate the means ± SEMs. Statistical significance was assessed using one-way ANOVA with multiple comparisons. **c** Representative images of brain sections from WT and knockout mice showing PLA signals to detect Tom20–Kif5b interactions (red) and nuclei (DAPI, blue). Dashed boxes indicate regions shown at higher magnification. Scale bars, 50 μm (upper panels), and 20 μm (lower panels). **d** Quantification of the normalized PLA signal intensity in the brain sections shown in **c** (*n* = 6 mice per group). Each dot represents one animal. The bars indicate the means ± SEMs. Statistical significance was assessed using an unpaired two-tailed *t*-test. **e**, **f** Co-IP assays of lysates from 293 T cells showing interactions between C19orf12 and mitochondrial transport proteins. 2×Strep-tagged C19orf12 (WT or A34V) was co-expressed with either 3×Flag–Miro1 (**e**) or 3×Flag–Kif5b (**f**). Input and immunoprecipitated samples were analyzed by immunoblotting with the indicated antibodies. **g** Immunoprecipitation of endogenous C19orf12 from U2OS cells harboring a 3×Flag–mScarlet–Miro1 knock-in was performed under DSP chemical cross-linking conditions. Input and immunoprecipitated samples were analyzed by immunoblotting to detect cross-linked and non-cross-linked forms of C19orf12 and associated proteins, as indicated. **h** Immunoprecipitation of 3×Flag–mScarlet–Miro1 U2OS KI cells performed in the presence of 1% n-dodecyl-β-D-maltoside (DDM), followed by an immunoblot analysis of co-precipitating C19orf12 and mitochondrial transport proteins. **i**, **j** In vitro GST pull-down assays using purified His–C19orf12–3×Flag and GST-tagged Miro1 (**i**) or Kif5b (**j**). Input and bound fractions were analyzed by immunoblotting with a Flag antibody. Coomassie blue staining indicates the loading of the purified proteins. The position of each purified protein band (including GST, GST-Miro1, and GST-Kif5b) is indicated by the asterisk adjacent to it.

The prominent perinuclear clustering of mitochondria observed in *C19orf12*-knockout cells suggests a specific deficit in anterograde transport. We therefore examined whether C19orf12 loss impairs the recruitment of the anterograde motor protein Kinesin-1 heavy chain (Kif5b) to mitochondria. Proximity ligation assay (PLA) revealed a marked reduction in Kif5b–Tom20 proximity signals in *C19orf12*-knockout U2OS cells and brain sections from *1600014C10Rik*-knockout mice (Fig. 5a–d). In contrast, Dynein–Tom20 proximity signals remained unchanged in *C19orf12*-knockout cells (Fig. 5a, b). Consistent with these findings, the knockdown of *1600014C10Rik* with two independent shRNAs reduced Kif5b–Tom20 PLA signals within the distal axons of primary mouse hippocampal neurons (Supplementary Fig. S6a, b). Together, these findings indicate that C19orf12 selectively facilitates the association of the anterograde motor machinery with mitochondria.

We performed affinity purification‒mass spectrometry (AP‒MS) using purified C19orf12 as bait to identify the molecular machinery engaged by C19orf12 (Supplementary Fig. S6c). This unbiased screen identified Kif5b and the mitochondrial Rho GTPase Miro1/2 as potential interacting partners of C19orf12 (Supplementary Fig. S6d).

We performed co-immunoprecipitation (co-IP) assays to validate the principal interactions and confirm their specificity, and the results showed robust interactions between C19orf12 and Miro1/2 as well as Kif5b (Fig. 5e, f; Supplementary Fig. S6e). Given the high sequence homology between the two Rho GTPases and the higher peptide coverage observed for Miro1, as well as previous reports of high Miro1 expression in several brain regions and its predominant role in long-range neuronal mitochondrial transport^48,49^, we focused our subsequent mechanistic characterization on Miro1. Notably, C19orf12 did not interact with the canonical mitochondrial adaptor TRAK1 (Supplementary Fig. S6f), indicating that it engages the transport machinery through a distinct, specific interface. Importantly, although the HSP-associated A34V mutant protein localized correctly to the ER (Fig. 3e), it exhibited a significantly reduced binding affinity for both Miro1 and Kif5b (Fig. 5e, f). This molecular defect — the inability to effectively recruit components of the motor complex — provides a mechanistic explanation for why the A34V variant fails to support mitochondrial transport and may contribute to the pathogenicity of A34V.

We utilized U2OS cells harboring a knock-in 3×Flag-Miro1 allele and captured the physiological C19orf12–Miro1 complex — along with known Miro1–Kif5b/TRAK assemblies — under cross-linking and mild detergent conditions to further confirm the interactions among the endogenous C19orf12, Miro1, and Kif5b proteins (Fig. 5g, h). Crucially, in vitro pull-down assays using purified recombinant proteins confirmed that C19orf12 acts as a direct physical linker by directly binding to both Miro1 and Kif5b (Fig. 5i, j). Detailed domain mapping using truncation mutants revealed distinct binding modes for these partners: C19orf12 specifically recognizes the C-terminal cargo-binding tail of Kif5b (amino acids 744–963) — the canonical site for adaptor attachment — whereas binding to Miro1 required the presence of multiple domains, which are likely to preserve the structural stability required for the interaction (Supplementary Fig. S6g–k).

### C19orf12 scaffolds the assembly of the Miro1–Kinesin-1 anterograde transport complex

**Fig. 6:**
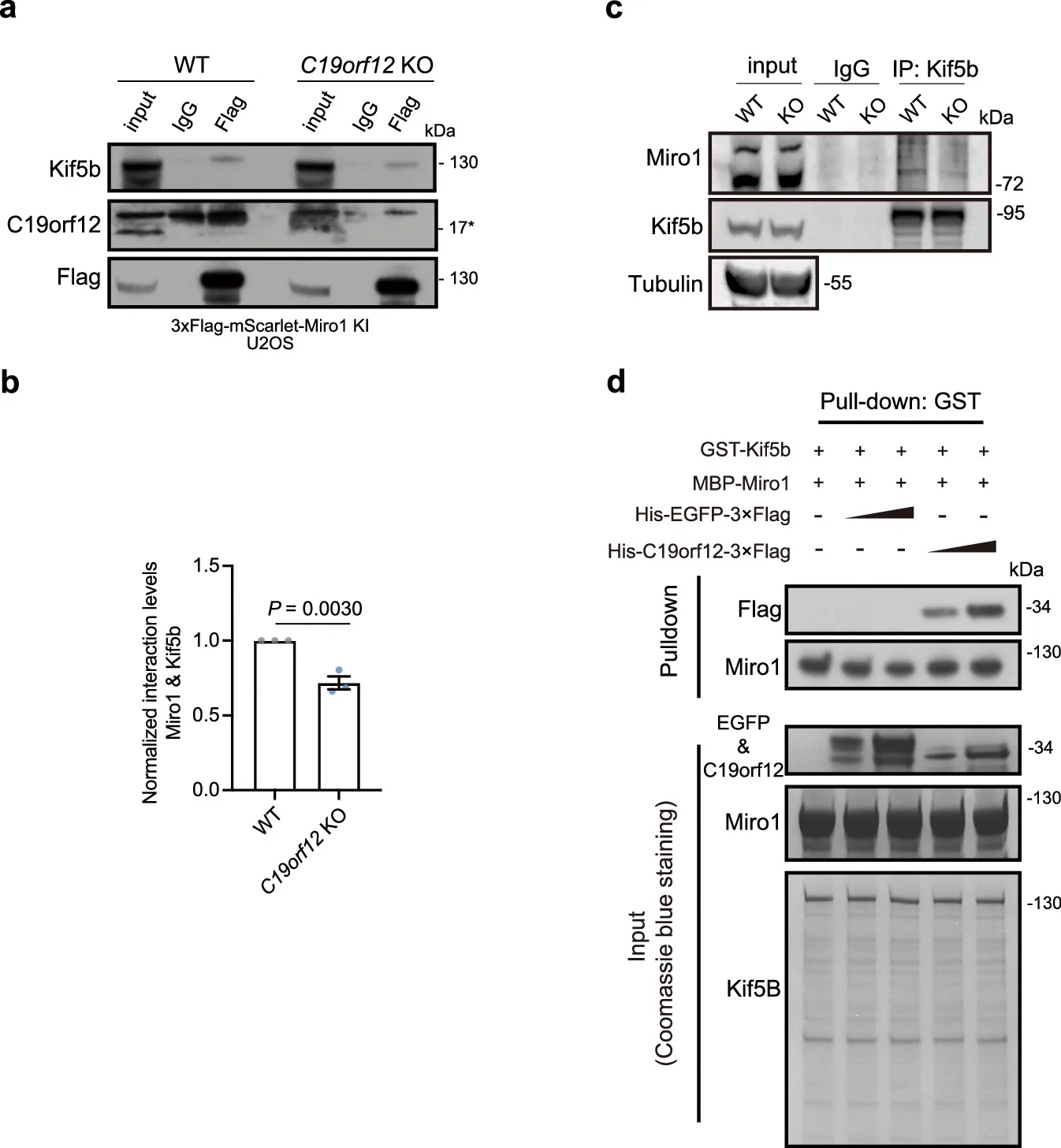
C19orf12 promotes the formation of the Miro1–Kif5b complex. **a** Immunoprecipitation of 3×Flag–mScarlet–Miro1 U2OS knock-in cells in which C19orf12 was subsequently deleted, followed by an immunoblot analysis of the co-precipitating Miro1 and Kif5b proteins. Input, IgG and anti-Flag immunoprecipitations are shown. **b** Quantification of the normalized interaction levels between Miro1 and Kif5b in WT and *C19orf12*-knockout cells, based on the data shown in **a** (*n* = 3 experiments). The data are shown as the means ± SEMs; statistical significance was assessed using an unpaired two-tailed *t*-test. **c** Co-IP of endogenous Kif5b from the brain lysates of WT and C19orf12-knockout mice, followed by immunoblotting for Miro1 and Kif5b. Input, IgG and anti-Kif5b immunoprecipitations are shown. **d** Competitive in vitro GST pull-down assays using GST–Kif5b and MBP–Miro1 in the presence of increasing amounts of purified His–C19orf12–3×Flag or His–EGFP–3×Flag as a control. Bound fractions were analyzed by immunoblotting for Flag and Miro1. Coomassie blue staining was performed to determine protein loading.

Having established that C19orf12 directly binds to components of the transport machinery, we investigated why its loss leads to motor protein detachment. Mechanistically, we hypothesized that C19orf12 acts as a scaffold to stabilize the Miro1–Kif5b complex. Consistent with these findings, the interaction between Miro1 and Kif5b was significantly reduced in *C19orf12*-knockout cells compared with WT control cells (Fig. 6a, b), and a similar reduction was observed in brain lysates from *1600014C10Rik*-knockout mice (Fig. 6c). Moreover, the results of in vitro binding assays revealed that the presence of C19orf12 promoted the interaction between Kif5b and Miro1 (Fig. 6d). Together, these data indicate that C19orf12 facilitates the efficient assembly of the Miro1–Kinesin-1 transport complex.

Given that C19orf12 does not physically bind to TRAK1 (Supplementary Fig. S6f), we investigated whether C19orf12 functions through independent mechanisms. The overexpression of TRAK1 is known to drive mitochondrial hyper-spreading^20,50^. TRAK1 overexpression induced mitochondrial extension in both WT and C19orf12-deficient cells to a similar extent (Supplementary Fig. S7a–c), indicating that TRAK1 can function independently of C19orf12 and does not require C19orf12 to promote mitochondrial redistribution under these overexpression conditions.

In contrast, we observed a distinct dependent relationship between C19orf12 and Miro1. While the overexpression of Miro1 induced a slight outward displacement of mitochondria and promoted hyper-fusion^16^, it failed to effectively restore the global mitochondrial distribution in *C19orf12*-knockout cells (Supplementary Fig. S7d–f). These findings indicate that increasing the Miro1 dosage alone is insufficient to drive long-range transport in the absence of C19orf12. We utilized *Miro1/2* double-knockout cells, which exhibit severe mitochondrial clustering^20,49^, to further clarify this dependent relationship. Reintroducing C19orf12 into these cells failed to rescue the clustering phenotype, confirming that C19orf12 cannot function independently of Miro1/2 (Supplementary Fig. S7g–i). Together, these results establish a mutually dependent relationship, with C19orf12 being essential for Miro1 to mediate efficient mitochondrial transport and Miro1/2 serving as the obligate mitochondrial partner for C19orf12 to scaffold the transport complex.

C19orf12 is dynamically associated with the ER membrane rather than being immobilized and transiently accumulates at ER‒mitochondria contact sites (Fig. 4). This dynamic pattern of enrichment is reminiscent of the behavior associated with biomolecular condensation. We therefore asked whether C19orf12 can undergo liquid‒liquid phase separation (LLPS), which could provide a mechanism for spatially concentrating components of the mitochondrial transport machinery. Under molecular crowding conditions, purified C19orf12 formed spherical condensates that underwent fusion and fission and displayed dynamic fluorescence recovery after photobleaching (FRAP), a behavior consistent with LLPS (Supplementary Figs. S8a,S9a, b). Neither Miro1 nor Kif5b formed condensates alone under the same conditions (Supplementary Fig. S8b, c), whereas both proteins were recruited into C19orf12-containing condensates (Supplementary Figs. S8b–d, S9c–g). These observations suggest that C19orf12 condensation may provide an additional mechanism for locally concentrating transport components and facilitating complex assembly.

### Ca^2+^ regulates C19orf12-mediated transport complex assembly and mitochondrial motility

Having established that C19orf12 facilitates the assembly of the Miro1–Kif5b complex, we next investigated how this activity might be regulated at ER–mitochondria contact sites. These contact sites coordinate several aspects of mitochondrial physiology, including lipid transfer, mitochondrial fission and fusion, and Ca^2+^ exchange^29–32^. Among these processes, Ca^2+^ signaling is particularly relevant because Miro1 contains Ca^2+^-sensing EF-hand motifs and is known to couple changes in cytosolic Ca^2+^ levels to mitochondrial motility^21,22^. Moreover, RNA sequencing of WT and *C19orf12*-knockout cells identified “calcium ion binding” as one of the most significantly upregulated functional categories in *C19orf12*-knockout cells (Supplementary Fig. S10), indicating altered Ca^2+^ homeostasis. We therefore assessed calcium‑related behaviors of the C19orf12‑containing transport machinery. In vitro pull‑down assays showed modest, non‑monotonic changes in C19orf12–Miro1 binding affinity across a range of free Ca^2+^ concentrations (Supplementary Fig. S11a, b). This calcium‑dependent modulation was largely blunted when the EF‑hand motifs of Miro1 were deleted (Supplementary Fig. S11c, d). Nonetheless, these effects were relatively mild in magnitude, and whether such calcium‑dependent shifts in binary C19orf12–Miro1 interactions occur under physiological conditions inside cells remains unclear.

Because the effect of Ca^2+^ on the binary interaction between C19orf12 and Miro1 was relatively limited, we next asked whether Ca^2+^ might exert a more pronounced effect on their higher-order organization. Purified C19orf12 formed droplets across the full range of Ca^2+^ concentrations examined. Measurements of droplet fluorescence and diameter revealed only limited variation, indicating that Ca^2+^ did not markedly affect the intrinsic condensation of C19orf12 alone (Supplementary Fig. S11e–g). In contrast, C19orf12–Miro1 co-condensates displayed a non-monotonic response to Ca^2+^. The number of droplets, the total fluorescence signals of the two proteins and the proportion of C19orf12 droplets containing Miro1 generally increased at low to intermediate Ca^2+^ concentrations but decreased in the presence of the highest Ca^2+^ concentration (Supplementary Fig. S11h–m). These observations suggest that Ca^2+^ may preferentially modulate the recruitment of Miro1 into C19orf12 condensates.

We next examined how local Ca^2+^ dynamics are related to mitochondrial motility using the mitochondrial surface-localized calcium sensor GCaMP6f–Tom5 (Supplementary Fig. S12a). Live‑cell time‑lapse imaging of mitochondrial surface calcium levels revealed a biphasic relationship between local mitochondrion‑associated Ca^2+^ signals and mitochondrial motility in neurons (Supplementary Fig. S12a, b). For the quantitative analysis, all the tracked mitochondria were ranked according to their relative surface Ca^2+^ signal and divided into five bins, with increasing bin numbers representing higher Ca^2+^ signals. The mitochondrial speed was relatively low in Bin 1, reached its maximum in Bin 2 and subsequently decreased as the relative Ca^2+^ signal increased across Bins 3–5 (Supplementary Fig. S12c). The progressive reduction in motility at higher Ca^2+^ levels is consistent with established models in which elevated Ca^2+^ levels promote mitochondrial arrest^24^. However, the reduced motility observed at the lowest Ca^2+^ level indicates that the relationship is biphasic rather than simply inhibitory.

## Discussion

**Fig. 7:**
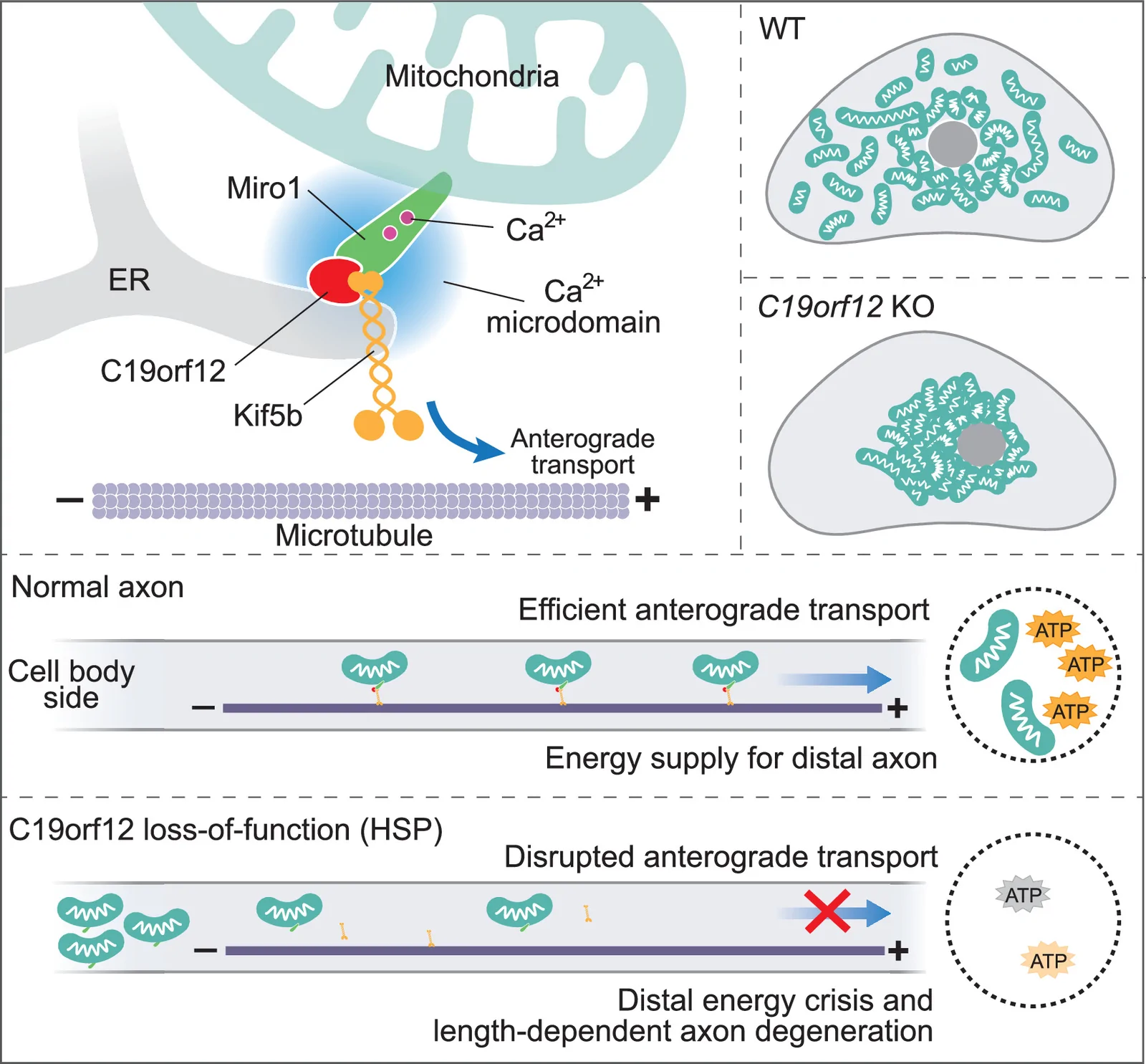
Proposed model for C19orf12-dependent mitochondrial transport and axonal maintenance. Proposed mechanism underlying the function of C19orf12 at ER–mitochondria contact sites (top left panel). Under physiological conditions, C19orf12 is dynamically associated with the ER membrane and is transiently enriched at ER–mitochondria contact sites. It functions as an ER-anchored scaffold that facilitates the assembly of the mitochondrial transport machinery by promoting the association between the mitochondrial outer-membrane protein Miro1 and the anterograde motor Kinesin-1 (Kif5b). Furthermore, the localized microdomains containing high Ca^2+^ levels at these contact sites provide the essential calcium required to support Miro1 activity. Cellular regulation of the mitochondrial distribution (top right panel). In WT cells (top panel), C19orf12 promotes the peripheral distribution of mitochondria. In the absence of C19orf12 (bottom panel), anterograde mitochondrial transport is impaired, leading to prominent mitochondrial perinuclear clustering. Neuronal consequences and HSP pathology (bottom panel). Under physiological conditions, C19orf12-mediated transport ensures the efficient delivery of mitochondria to distal axonal terminals, maintaining the local ATP availability required for synaptic homeostasis. Under pathological conditions, the failure of C19orf12-dependent transport leads to the progressive depletion of mitochondria at the distal axon. The resulting reduction in local ATP availability is proposed to contribute to the “dying-back”, length-dependent axonopathy characteristic of HSP.

Our study identifies C19orf12 as an ER-anchored regulator that orchestrates mitochondrial transport by scaffolding the Miro1–Kif5b complex (Fig. 7), expanding the functional dimension of ER–mitochondria contact sites. The loss of C19orf12 in mice causes progressive spasticity, muscle weakness, and gait instability, robustly recapitulating the SPG43 and NBIA phenotypes^40,51^. At the cellular level, impaired anterograde mitochondrial transport creates a “supply chain crisis” in long axons: while the soma retains clustered mitochondria, distal axons suffer from a severe ATP depletion. This spatial bioenergetic mismatch directly links the molecular defect in C19orf12 to the “dying-back” vulnerability of distal synapses characteristic of HSP and NBIA^8,37^.

Addressing ambiguities from previous overexpression studies^40^, our endogenous tagging and single-molecule tracking assays reveal that physiological C19orf12 predominantly associates with the ER membrane and is concentrated transiently at ER–mitochondria contacts. Crucially, chimeric rescue experiments demonstrated that ER anchoring — but not mitochondrial outer membrane targeting — is strictly required for C19orf12 function. However, ER localization alone is insufficient: the pathogenic A34V variant correctly targets the ER but fails to bind the Miro1–Kif5b machinery and cannot restore the mitochondrial distribution. These findings suggest that defective effector engagement and motor protein recruitment contribute to the pathogenesis of C19orf12-associated SPG43 and NBIA even when ER targeting is preserved.

In addition to stabilizing Miro1–Kif5b interactions, our in vitro observations showed that purified C19orf12 forms dynamic condensates that recruit Miro1 and Kif5b. This property may provide a plausible mechanism for the local accumulation of transport machinery components. In support of this hypothesis, an independent study recently reported that C19orf12 condensation facilitates BNIP3-mediated neuronal mitophagy^52^. These complementary findings suggest that condensation may represent a broader organizational property through which C19orf12 coordinates distinct mitochondrion-related processes.

Our biochemical mapping results revealed that C19orf12 is associated with the Kinesin-1/Miro1 machinery but not with the adaptor TRAK1. This distinction is functionally relevant: TRAK1 overexpression promoted mitochondrial redistribution in C19orf12-deficient cells, whereas Miro1 overexpression alone was insufficient to restore the normal mitochondrial distribution. These observations suggest that the canonical Miro–TRAK–Kinesin pathway and the C19orf12-facilitated mode of motor protein recruitment are complementary rather than mutually exclusive. In a complementary study by our group, ATL2 was shown to recruit TRAK1 to the ER and promote TRAK1–Miro1 assembly at ER–mitochondria contact sites^53^. This ATL2-dependent route provides a potential mechanism by which TRAK1 overexpression can bypass C19orf12 deficiency, whereas C19orf12 promotes Miro1–Kif5b assembly without directly engaging TRAK1.

An important question is why C19orf12 must be anchored to the ER. As the major intracellular Ca^2+^ store, the ER generates local Ca^2+^ microdomains at contact sites^26,27^. While an elevated cytosolic Ca^2+^ level is a well-established “stop signal” for Miro1-dependent transport^21,22,54^, recent reconstitution experiments showed that Ca^2+^ did not directly inhibit the motility of Miro1–TRAK–Kinesin complexes, implicating additional factors such as Syntaphilin-mediated anchoring in mitochondrial arrest^55^. C19orf12 may contribute to such context-dependent regulation at ER–mitochondria interfaces. Our live-cell imaging revealed a biphasic relationship: mitochondrial motility was restricted to both the lowest and highest Ca^2+^ concentrations but peaked at intermediate levels (Supplementary Fig. S12). This result suggests that the local Ca^2+^ concentration acts as a rheostat rather than exclusively as an inhibitory signal. Consistent with this finding, in vitro Ca^2+^ concentrations had a modest, non-monotonic effect on the binary C19orf12–Miro1 interaction that depended on the EF hand domains of Miro1 (Supplementary Fig. S11a–d). More markedly, variations in the Ca^2+^ level tuned the recruitment of Miro1 into higher-order C19orf12 condensates (Supplementary Fig. S11e–m). Although these assays do not establish direct motor regulation in cells, they suggest that ER-derived Ca^2+^ microdomains may fine-tune the composition of C19orf12–Miro1 assemblies, adding a context-dependent regulatory layer to mitochondrial motility.

In summary, we define C19orf12 as an essential ER-based regulator that couples the mitochondrial transport machinery to the ER network. Our findings not only clarify the pathogenic mechanism of SPG43 but also highlight the ER–mitochondria interface as a critical command center for the logistic support of long axons.

## Materials and methods

### Animals

C57BL/6J mice (6–8 weeks old) were obtained from Beijing Vital River Laboratory Animal Technology Co., Ltd. (Beijing, China). *1600014C10Rik*-knockout mice were generated by Cyagen Biosciences using CRISPR/Cas9 technology. The following sgRNAs targeting the murine *1600014C10Rik* gene were designed: forward 5′-AAATGATTATGAGTCCAGAC-3′ and reverse 5′-TGGCGGCTTACAATCAATGC-3′. F0 heterozygous mice were backcrossed to WT C57BL/6J mice for two generations, and F3 heterozygotes were intercrossed to generate homozygous knockout mice. All mice were housed in specific pathogen-free facilities at the Experimental Animal Center of Peking University. All animal procedures were conducted in strict accordance with protocols approved by the Institutional Animal Care and Use Committee of Peking University.

### Mouse behavioral tests

All behavioral tests were performed on age-matched mice that were trained 24 h prior to testing.

Hindlimb clasping: mice were held by their tails for 10 s and scored on a scale of 0–3 points based on the degree of splaying outward from the abdomen: 0, the hindlimbs are fully spread and moving; 1, > 50% retraction of one hindlimb toward the abdomen; 2, > 50% retraction of both hindlimbs toward the abdomen; and 3, both hindlimbs were fully retracted to the abdomen.

Balance beam: mice were trained to walk across a horizontal beam (100 cm long, 12 mm wide, and 50 cm tall) to their home cages, and beam-walking was recorded using a video camera. Each mouse was tested three times separately.

Forelimb grip: the mouse was placed over the grid with its forepaws grasping the grid. The mouse was gently pulled backward by its tail to ensure that it held on to the upper part of the grid while a horizontal position of the torso was maintained. The maximum grip strength value displayed on the screen was recorded. Each mouse was tested three times separately.

Pole test: mice were positioned at the top of a vertical pole (50 cm tall and 1 cm in diameter) and assessed for their ability to descend the pole (with the snout leading) by measuring the time from the initiation of the head-down movement until all four paws reached the platform. Each mouse was tested three times separately.

Rotarod test: mice were placed on the rod rotating at 4 rpm for habituation and then the speed of rotation of the rotarod was accelerated from 4 to 40 rpm in 5 min. The time until the mice fell off the rotarod was recorded. Each mouse was allowed a 10 min interval between trials and was tested three times per day for two consecutive days.

Gait analysis: the mice were gently grasped, and the paws were painted; one color was used for the hind limbs, and another was used for the forelimbs. The mice were allowed to walk on paper inside a tunnel (35 cm long, 7 cm wide, and 8 cm tall), and a sufficient number of steps (more than four) were collected. The footprints created by the same foot were counted to calculate the stride width and length. Five steps were counted to calculate the average distance traveled.

### Plasmids and molecular cloning

The plasmid used for ATP imaging, FRET-based ATP biosensor ATeam1.03-nD/nA/pcDNA3 (Addgene plasmid, #51958), was a gift from Takeharu Nagai. The DNA fragments were amplified by PCR from cDNA or genomic DNA templates and cloned and inserted into vectors using 2X MultiF Seamless Assembly Mix (ABclonal, RK21020). Briefly, the sequences encoding the WT and corresponding mutant human C19orf12 proteins, Miro1, Miro2 and Kif5b were cloned and inserted into the vectors pSIN-3×Flag-mCherry-IRES-puro, p3×FLAG-CMV-7.1 (Sigma‒Aldrich, E7533), p3×FLAG-CMV-14 (Sigma‒Aldrich, E7908), pcDNA3.1 (+) (Invitrogen, V79020), pCold-MBP (NovoPro Bioscience Inc., V012984), pET-28a (+) (Novagen, 69864), or pGEX-6P-1 (GE Healthcare, 28-9546-48). A schematic of the truncation constructs of C19orf12 is shown in Fig. 4f. Chimeric mutants of C19orf12 were generated based on the following amino acid boundaries: C19orf12 pTM (44–81 aa) was replaced with ATL3_TM of ATL3 (446–488 aa), EI24_TM of EI24 (79–138 aa) and MFN2_TM of MFN2 (601–651 aa). Schematics of the truncated constructs of Kif5b and Miro1 are shown in Supplementary Fig. S6g, j. The plasmid used for calcium imaging was edited on the backbone of pGP-CMV-GCaMP6f (Addgene plasmid, #40755) by fusing the sequence with the mitochondrial outer membrane protein Tom5. Plasmids used for knockout and knockdown were edited on the backbone of pSpCas9(BB)-2A-Puro (PX459) V2.0 (Addgene plasmid, #62988), lentiCRISPR v2 (Addgene plasmid, #52961), and pLKO.1 puro (Addgene plasmid, #8453). All plasmid sequences were verified using Sanger sequencing (RuiBiotech).

### Antibodies

The following primary antibodies were used: rabbit polyclonal anti-C19orf12 (Proteintech, 27382-1-AP; immunoblotting, 1:500), mouse monoclonal anti-Miro1 (Sigma‒Aldrich, WH0055288M1; immunoblotting, 1:1000), rabbit polyclonal anti-Miro2 (Proteintech, 11237-1-AP; immunoblotting, 1:1000), rabbit polyclonal anti-TRAK1 (Sigma–Aldrich, HPA005853; immunoblotting, 1:1000), rabbit polyclonal anti-TRAK2 (Proteintech, 13770-1-AP; immunoblotting, 1:1000), rabbit polyclonal anti-Kif5b (Proteintech, 21632-1-AP; immunoblotting, 1:2000; immunofluorescence staining, 1:200), rabbit polyclonal anti-MFN1 (Proteintech, 13798-1-AP; immunoblotting, 1:1000), rabbit polyclonal anti-MFN2 (Proteintech, 12186-1-AP; immunoblotting, 1:1000), rabbit polyclonal anti-KLC1 (Proteintech, 19028-1-AP; immunoblotting, 1:1000), rabbit polyclonal anti-KLC2 (Proteintech, 17668-1-AP; immunoblotting, 1:1000), rabbit polyclonal anti-KLC3 (Proteintech, 11645-1-AP; immunoblotting, 1:1000), rabbit polyclonal anti-KLC4 (Proteintech, 16493-1-AP; immunoblotting, 1:1000), mouse monoclonal anti-Tom20 (BD Biosciences, 612278; immunofluorescence staining, 1:500), rabbit polyclonal anti-Tom20 (Proteintech, 11802-1-AP; immunoblotting, 1:5000, immunofluorescence staining, 1:1000), mouse monoclonal anti-LAMP1 (Santa Cruz, sc-20011; immunoblotting, 1:500; immunofluorescence staining, 1:200), rabbit polyclonal anti-Calnexin (Proteintech, 10427-2-AP; immunoblotting, 1:5000), rabbit polyclonal anti-Cytochrome C (Proteintech, 10993-1-AP; immunoblotting, 1:1000), mouse monoclonal anti-Flag (Sigma–Aldrich, F1804, clone M2; immunoblotting, 1:5000; immunofluorescence staining, 1:500), rabbit polyclonal anti-mCherry (Proteintech, 26765-1-AP; immunoblotting, 1:1000), mouse monoclonal anti-α-Tubulin (Sigma–Aldrich, T5168, clone B512, immunoblotting, 1:5000; immunofluorescence staining, 1:1000), mouse monoclonal anti-GAPDH (Proteintech, 60004-1-Ig; immunoblotting, 1:20000), rabbit polyclonal anti-Calreticulin (Proteintech, 27298-1-AP; immunoblotting, 1:50000), rabbit polyclonal anti-Dynein (Abcam, ab111177; immunofluorescence staining, 1:200), and rabbit polyclonal anti-GFP (Proteintech, 50430-2-AP; immunoblotting, 1:3000). Horseradish peroxidase-conjugated goat anti-rabbit or anti-mouse secondary antibodies were purchased from Jackson ImmunoResearch. Alexa Fluor 488/568/647-conjugated goat anti-mouse or anti-rabbit IgG (H + L) highly cross-adsorbed secondary antibodies were obtained from Invitrogen.

### Cell culture

U2OS, HeLa, HEK-293T, and COS7 cells were cultured in Dulbecco’s modified Eagle’s medium (DMEM; CellMax, CGM101.06) supplemented with 10% (v/v) fetal bovine serum (FBS), and A549 cells were cultured in Ham’s F-12K (Kaighn’s) medium (Meiluncell, PWL011) supplemented with 10% FBS. All the media described above were supplemented with penicillin (100 units/mL) and streptomycin (100 μg/mL). Primary hippocampal neurons isolated from embryonic day 16.5 (E16.5) mouse brains were cultured in Neurobasal-A medium (Gibco, 10888022) supplemented with 2% (v/v) B-27 (Gibco, 17504-044) and 1% (v/v) GlutaMAX (Gibco, 35050-061). All the cells were maintained at 37 °C in a humidified incubator with 5% CO_2_. For subculture, cells were dissociated with 0.1% trypsin, suspended by pipetting, and pelleted by low‑speed centrifugation, followed by re‑plating at the desired density. Cryopreservation was performed in a freezing solution containing DMSO and FBS (1:9, v/v), and the cells were stored in liquid‑phase liquid nitrogen.

### Primary mouse hippocampal neuron culture

All procedures were performed under sterile conditions. Briefly, glass coverslips or dishes were coated with poly‑D‑lysine (1 mg/mL in borate buffer, pH 8.5) overnight at 37 °C in a humidified chamber, washed twice with sterile ddH_2_O (2 h each), and equilibrated with plating medium (DMEM supplemented with 10% FBS) overnight. Dissection was performed in ice‑cold dissociation medium (136.9 mM NaCl, 5.4 mM KCl, 4.2 mM NaHCO_3_, 0.4 mM KH_2_PO_4_, 0.4 mM Na_2_HPO_4_, 5.6 mM glucose, 1 mM sodium pyruvate, and 10 mM HEPES, pH 7.4). Hippocampal tissues were isolated from E16.5 mice, digested with papain at 37 °C for 18–20 min, treated with 50 µL of 10 mg/mL DNase I, and mechanically dissociated by gentle pipetting in plating medium. The resulting cell suspension was centrifuged at 500× *g* for 2 min, resuspended, and filtered through a 70‑µm strainer. Cells were plated onto coated coverslips at 5 × 10^4^ cells per coverslip in 100–150 µL of plating medium. After 4–5 h, the medium was replaced with culture medium (Neurobasal medium supplemented with 2% B‑27 and 1% GlutaMAX). The entire dissection was completed within 2 h.

### Transfection and lentiviral transduction

For HEK293T cells, transfections were performed using polyethyleneimine (PEI; Polysciences, 23966-1) at 60% confluency. The medium was replaced 6 h post-transfection, and the cells were harvested after 24 h. For the other cell lines, transfections were performed using Omni transfection reagent (EZ Biosystems, EZT-OMNI). For lentivirus production, HEK293T cells were transfected with three plasmids, including two packaging plasmids, psPAX2 (Addgene plasmid, #12260) and pVSV-G (Addgene plasmid, #138479), and the indicated lentivirus plasmid. After 6 h, the medium was replaced with DMEM containing 20% FBS. Viral supernatants were collected 48–72 h post-transfection, filtered, and used to infect target cells. The cell culture supernatant was mixed with a one-third volume of a PEG 8000–NaCl solution (0.4 g/mL PEG 8000 (Amresco, 97061-098) and 1.2 M NaCl in phosphate-buffered saline (PBS), pH 7.4) at 4 °C overnight with end-over-end mixing and centrifuged at 1800× *g* for 20 min at 4 °C to determine the lentivirus concentration. The obtained supernatant was discarded, and the pellet containing lentivirus was dissolved in PBS or DMEM. Primary mouse neurons were infected at DIV 3 and analyzed at DIV 8.

### Generation of knockout, knock-in and knockdown cell lines

Gene-edited cell lines were generated using CRISPR/Cas9. The following sgRNA sequences were used for the knockout cell lines:

human *C19orf12* (5′-TGGGGAGAGGAAGATGAAGG-3′);

human *Miro1* (5′-TCTGGTGGAATATAGTAGTA-3′);

human *Miro2* (5′-ACAAGGTACCCGTGGTGCGC-3′);

human *TRAK1* (5′-GAATGTTGTACTTCGATCCG-3′).

For the knock-in lines, donor templates were designed to tag endogenous *C19orf12* (C-terminal Halo-3×Flag-ALFA, 5′-TGGGGAGAGGAAGATGAAGG-3′) or *Miro1* (N-terminal 3×Flag-mScarlet, 5′-TTCTTCATGTCGGCGGCTCT-3′). A549 *C19orf12*-knockdown cells were generated using an siRNA (5′-GCAACAGAGGCTCTTTAACGA-3′) targeting the *C19orf12* transcript. Mouse *1600014C10Rik*-knockdown neurons were generated using shRNA#1 (5′-TGGGAAACTTGTCTAATACCAGCCTC-3′) and shRNA#2 (5′-CTCTTGAGTAACTTTAGTTTAGCCTC-3′).

### Immunofluorescence staining and live-cell imaging

For immunofluorescence staining, the cells were fixed with 4% paraformaldehyde in PBS for 30 min at 37 °C and permeabilized with 0.1% Triton X-100 in PBS for 10 min. After blocking with 4% BSA for 30 min, the cells were incubated with the primary antibodies at 37 °C for 2 h and subsequently incubated with the secondary antibodies in the dark at 37 °C for 1 h, followed by three washes with PBS. Coverslips were mounted using a glycerol/PBS (1:1) solution containing DAPI and stored at 4 °C. Imaging was performed using a Zeiss LSM980 microscope equipped with a 63×/1.4 oil objective lens in Airyscan mode. Images were acquired using ZEN software (Carl Zeiss) and processed with ZEN software or Fiji.

For live-cell imaging, cells cultured in glass-bottom dishes were maintained at 37 °C in a 5% CO₂ atmosphere. Images were acquired using a Zeiss LSM980 confocal microscope equipped with a 63×/1.4 oil‑immersion objective in Airyscan mode.

### Imaging and quantification of mitochondria in the axons of hippocampal neurons

Primary hippocampal neurons were infected with lentivirus carrying shRNAs (shCtrl, sh*1600014C10Rik* #1, or sh*1600014C10Rik* #2) at DIV 3 and imaged at DIV 8. The mitochondria were labeled with PKMito (200 nM, 37 °C, 15 min). For the motility analysis, time‑lapse images were acquired every 5 s for 5 min. A 30‑µm segment at the distal axon was selected for quantification. Kymographs were generated using Fiji software. The mitochondria that exhibited obvious displacement during the acquisition period were scored as motile, and the percentage of motile mitochondria was calculated. The same 30‑µm distal axonal segment was analyzed by measuring the mitochondrial fluorescence intensity using Fiji to determine the mitochondrial density in the distal axon.

### ATP imaging using ATeam1.03

Neurons were transfected with the FRET-based ATP biosensor ATeam1.03-nD/nA/pcDNA3. Cells were imaged using a Zeiss LSM980 microscope equipped with a 405-nm laser for CFP excitation. Dual-emission images were acquired for CFP (460–500 nm) and YFP (520–550 nm). Images were analyzed using Fiji software. The background was subtracted from both channels, and the pixel-by-pixel ratio of YFP/CFP was calculated to generate pseudocolor ratio images.

### JC-1 staining

Cells cultured on chamber slides were stained with a 10 μg/mL JC-1 working solution at 37 °C in a 5% CO_2_ incubator for 15 min. After washes with PBS, the cells were cultured in complete medium for live-cell imaging using a Zeiss LSM980 microscope equipped with a 63×/1.4 oil objective lens in confocal mode. Images were acquired using ZEN software (Carl Zeiss) and processed with ZEN software or Fiji.

### SMT

U2OS C19orf12–Halo knock-in cells were transiently transfected with EGFP–Sec61β to label the ER. Before imaging, the endogenous C19orf12–Halo protein was labeled with the HaloTag ligand BD566HTL^56^, and mitochondria were stained with PKMito Deep Red^57^. Following labeling, the cells were subjected to live-cell imaging.

SMT was performed using a custom-built microscope based on a Nikon Ti2 body equipped with an AST micro- and nano-positioner. Illumination and the capture of fluorescence images were performed using a Nikon 100×/1.49 NA APO total internal reflection fluorescence (TIRF) SR oil-immersion objective. Excitation was provided by a 642-nm laser (2RU-VFL-P-1000-642-B1R, 2 W; MPB Communications), a 561-nm laser (2RU-VFL-P-1000-561-B1R, 2 W; MPB Communications), and a 488-nm laser (OBIS, 200 mW; Coherent). A full-multiband filter set (ZET405/488/561/640mv2; Chroma) was used, and excitation was performed under TIRF illumination. Emitted fluorescence was directed to a W-VIEW GEMINI-2C image-splitting module (Hamamatsu) and recorded using a Prime 95B sCMOS camera (Photometrics; 88% quantum efficiency at 670 nm).

Unless indicated otherwise, each imaging cycle consisted of one 100-ms exposure in the 488-nm channel and one 100-ms exposure in the 561-nm channel, followed by 250 consecutive 10-ms exposures in the 640-nm channel under TIRF illumination. The illumination power was 100 mW for the 488-nm and 561-nm channels and 300 mW for the 640-nm channel.

### PLA

PLA (Sigma‒Aldrich, DUO92101) was performed according to the manufacturer’s instructions. Briefly, samples were prepared and incubated with the primary antibody according to the immunofluorescence staining protocol described above. After washes with Wash Buffer A, the samples were incubated with PLUS and MINUS PLA probes for 1 h at 37 °C. The probes were subsequently ligated for 30 min at 37 °C and amplified for 100 min at 37 °C after the samples were washed with Wash Buffer A. After washes with Wash Buffer B, the coverslips were mounted with Duolink® PLA Mounting Medium supplemented with DAPI. Samples were observed under a Zeiss LSM980 confocal microscope using the Airyscan function. The total intensity of the PLA signal per cell was quantified using Fiji software.

### Neuropathological analysis

Brain tissues were collected from adult mice after anesthesia induction using isoflurane (RWD, R510-22-10) and perfused with ice-cold PBS to flush the blood and blood cells from the brain, followed by perfusion with a 4% PFA solution for fixation. The fixed samples were sent to the pathology center for paraffin sectioning, and the resulting paraffin sections were subjected to immunofluorescence staining. After dewaxing with xylene for 10 min twice, the paraffin sections were rehydrated with different concentrations of ethanol (100%–95%–80%–50%–25%) for 5 min each. The sections were then washed with water and boiled in 100 mM sodium citrate buffer (pH 6.0) for 10 min twice for antigen retrieval, followed by a 20-min incubation at room temperature and washes with water and PBS. The subsequent permeabilization and blocking steps were the same as those used for cellular immunofluorescence staining.

### Mitochondrial calcium imaging

The GCaMP6f sequence was modified by fusing it with the mitochondrial outer membrane protein Tom5 to generate GCaMP6f‑Tom5, which directed the probe to the outer mitochondrial membrane, to monitor mitochondrial surface calcium dynamics. Primary mouse hippocampal neurons were infected with GCaMP6f‑Tom5 lentivirus at DIV 3 and imaged at DIV 8. Before imaging, the mitochondria were labeled with PKMito^57^ (200 nM, 37 °C, 15 min). Time‑lapse imaging was performed on a Zeiss LSM980 confocal microscope, with one frame acquired every 4 s over a total period of 5 min per field of view. For data analysis, the acquired image stacks were processed using IMARIS software. The surface module was employed to automatically delineate mitochondrial contours after manual selection, and trajectory data for individual mitochondria were generated. The X‑Y coordinates and GCaMP6f signal intensity were extracted for each frame. The speed of mitochondrial movement was calculated from the differences in the positions of the mitochondria between consecutive frames and correlated with the calcium signal intensity to generate a line graph of the speed versus the relative calcium signal.

### Western blotting

Cells were lysed with loading buffer (50 mM Tris, pH 6.8, 1 mM DTT, 10% glycerol, 2% SDS, 0.1% bromophenol blue) and boiled at 100 °C for 10 min. Samples were separated by SDS‒PAGE (GenScript) and transferred to PVDF membranes. The membranes were blocked with 4% milk in TBST (20 mM Tris (pH 7.4), 150 mM NaCl, and 0.1% Tween-20) and incubated with the primary antibody at 4 °C overnight. After washes with TBST, the membranes were incubated with the secondary antibody at room temperature for 2 h, after which they were washed with TBST. Signals were detected using SuperSignal Peroxidase Solution (Biosciences, TE0015-2) and captured on X-ray film (FUJIFILM) or ePHOTO (GenScript). Band intensities were quantified using Fiji software.

### RNA extraction and quantitative real-time PCR (qPCR) assay

Total RNA was extracted from cells or tissues using an RNA Easy Fast Tissue/Cell Kit (TIANGEN; DP451) according to the manufacturer’s instructions. For reverse transcription, 2 μg of total RNA was reverse-transcribed into complementary DNA (cDNA) using a Hifair® AdvanceFast 1st Strand cDNA Synthesis SuperMix for qPCR (DNA digester plus) Kit (YEASEN, 11155ES60) according to the manufacturer’s protocol. qPCR was performed using Hieff UNICON® Advanced qPCR SYBR Master Mix (YEASEN, 11185ES08) on a LightCycler® 96 (Roche). The following primers were used: *Hprt1*, forward 5′-TCAGTCAACGGGGGACATAAA-3′ and reverse 5′-GGGGCTGTACTGCTTAACCAG-3′; *1600014C10Rik*, forward 5′-TCCAGCTCGGTAAAGGGTC-3′ and reverse 5′-AACAGCTATTCCTGGTGGGC-3′. Relative gene expression was calculated using the 2^–ΔΔ*Ct*^ method and normalized to that of the endogenous reference gene *Hprt1*. All reactions were performed in triplicate.

### RNA-seq analysis

Total RNA was extracted from U2OS WT cells and two independent *C19orf12*-knockout clones using an RNA Easy Fast Tissue/Cell Kit (TIANGEN; DP451) according to the manufacturer’s instructions. For each cell line, three biological replicates were subjected to RNA‑seq. mRNA library construction and sequencing were performed using GENEWIZ from Azenta Life Sciences (Suzhou, China). Libraries with different indices were multiplexed and loaded in an Illumina NovaSeq instrument for sequencing using a 2 × 150 paired-end configuration according to the manufacturer’s instructions. Raw sequencing reads in FASTQ format were processed using Fastp (v0.24.1) to remove technical sequences and low-quality bases. Clean reads were aligned to the *Homo sapiens* genome (GRCh38.107) using HISAT2 (v2.2.1; ^58^). Gene-level counts were quantified using HTSeq (v0.6.1). Reads overlapping exons were counted, and multiple mapping reads or reads overlapping multiple genes were excluded. Differentially expressed genes (DEGs) were identified using DESeq2 (v1.34.0), and genes whose |log_2_ fold change| was ≥1 and whose FDR was < 0.05 were classified as differentially expressed.

### Measurement of the intracellular ATP concentration

The intracellular ATP concentration (Beyotime, S0026) was measured according to the manufacturer’s instructions. Briefly, cells were lysed on ice and centrifuged at 12,000× *g* for 5 min at 4 °C, after which the supernatant was collected for subsequent assays. ATP standard solutions were prepared by diluting the ATP standard with the assay lysis buffer to appropriate concentrations. For ATP measurements, 100 μL of the working solution was added to each well and incubated at room temperature for 5 min to deplete the background ATP. Then, 20 μL of sample or standard was added and mixed rapidly by pipetting, and after a minimum interval of 2 s, the relative luminescence units (RLUs) were measured using a luminometer. The ATP concentration in each sample was calculated from the standard curve. The protein concentration of the same lysate was determined using a BCA protein assay kit, and ATP levels were normalized to nmol per mg of protein to correct for variations in the protein content.

### Measurement of ROS production

ROS production was measured using CellROX® Oxidative Stress Reagents (Invitrogen, C10422). Briefly, CellROX® deep red reagent was added directly to the medium to a final concentration of 5 μM, and the cells were incubated at 37 °C for 30 min. After the medium was removed, the cells were washed three times with PBS. The fluorescence intensity of each sample was measured using a flow cytometer (CytoFlex LX Mosaic88).

### Subcellular fractionation

The isolation of subcellular fractions was conducted as previously described^47^. If cross-linking was needed, the following modification to the protocol was applied: the cells were incubated with 1 mM dithiobis (succinimidyl propionate) (DSP) (Thermo Scientific, PG82081) at 37 °C for 30 min, and the reaction was quenched by the addition of 10 mM Tris buffer. After collection and homogenization, the cells were centrifuged for 5 min at 600 × *g* twice to remove whole cells and nuclei. The supernatant was collected and centrifuged for 10 min at 7000 × *g* twice, and the pellet contained the crude mitochondria, while the supernatant contained the plasma membrane, lysosomes, microsomes and cytosol. The cytosolic supernatant was centrifuged for 30 min at 20,000 × *g*, and the supernatant was collected and further centrifuged for 1 h at 100,000 × *g*. The pellet contained the ER while the supernatant contained the cytosol. The crude mitochondrial pellet was suspended and centrifuged for 10 min at 10,000 × *g* to remove contaminants and obtain pure mitochondria.

The crude mitochondrial fractions were resuspended in mitochondrial resuspension buffer and layered on top of Percoll medium. Following centrifugation at 95,000 × *g* for 30 min, the crude MAM fraction appeared as a diffuse white band near the top of the medium, while a dense band containing purified mitochondria settled near the bottom of the tube. Both the MAM and pure mitochondrial fractions were further centrifuged to eliminate the Percoll. All the fractions obtained were rapidly frozen using liquid nitrogen and stored at −80 °C until they were ready to be used.

### Immunoprecipitation

Cells were lysed in lysis buffer (50 mM Tris-HCl, 150 mM NaCl, 1 mM EDTA, 0.5% Triton X-100, pH 7.5) containing the protease inhibitor PMSF and a protease inhibitor cocktail on ice for 30 min. The cell lysates were centrifuged at 12,500 rpm for 10 min, and the supernatants were collected as input and for immunoprecipitation by mixing them with antibody-conjugated Protein A/G beads for 2 h at 4 °C. The beads were washed five times with lysis buffer, and the bound proteins were eluted in sample buffer at 100 °C for 10 min for immunoblotting. Where indicated, an additional cross-linking step was incorporated into the protocol prior to cell lysis. Specifically, the cells were incubated with 1 mM DSP at 37 °C for 30 min, followed by quenching with 10 mM Tris buffer. All other steps were the same as the standard protocol described above.

### Mass spectrometry analysis of C19orf12

For mass spectrometry, HEK293T cells expressing Strep-tagged C19orf12 or mouse brains (*n* = 10) were lysed in appropriate buffers (cell lysis: 50 mM Tris-HCl (pH 7.5), 750 mM NaCl, 1% Triton X-100, and 0.1 mM biotin; brain tissue lysis: 50 mM Tris-HCl (pH 7.5), 150 mM NaCl, 0.1% SDS, 0.5% sodium deoxycholate, and 1% Triton X-100). The lysates were centrifuged at 15,000 rpm at 4 °C for 15 min, after which the supernatants were collected separately. The cell lysate supernatant was then incubated with Strep-Tactin beads for 2 h at 4 °C, followed by three washes with Wash Buffer 1 (50 mM Tris, 750 mM NaCl, 1% Triton X-100, pH 7.5). Mouse brain lysate supernatants were subsequently incubated with the beads for 2 h at 4 °C. Following the incubation, the beads were washed three times with Wash Buffer 1 and twice with Wash Buffer 2 (50 mM Tris, 150 mM NaCl, pH 7.5) to remove high-salt contaminants. After the supernatant was discarded, loading buffer was added to the beads. The eluted proteins were separated by SDS‒PAGE, stained with Coomassie blue, and excised for the LC‒MS/MS analysis.

### Prokaryotic expression, purification and in vitro binding assays

GST-Kif5b, GST-Miro1, C19orf12-6×His and MBP-Miro1 proteins were expressed in *E. coli* BL21 (DE3) induced with 1 mM IPTG at 16 °C for 12–16 h. The cells were lysed by sonication in GST lysis buffer (50 mM Tris-HCl, 250 mM NaCl, 0.5% Triton X-100, pH 8.0), His lysis buffer (50 mM Tris-HCl, 300 mM NaCl, 0.5% Triton X-100, 2 mM β-mercaptoethanol and 20 mM imidazole, pH 8.0), and MBP lysis buffer (20 mM Tris-HCl, 200 mM NaCl, 1 mM EDTA, 1 mM DTT (optional), pH 7.5), followed by centrifugation at 13,500 rpm for 15 min at 4 °C. The supernatant was collected and incubated with the corresponding affinity beads (GST-Sepharose, Ni-NTA, or Amylose resin) at 4 °C with rotation for 2 h. The beads were subsequently washed five times with wash buffer (50 mM Tris-HCl, 450 mM NaCl, 0.5% Tween-20, pH 7.5), and the C19orf12-6×His was eluted using His elution buffer (50 mM Tris-HCl, 300 mM NaCl, 0.5% Triton X-100, 2 mM β-mercaptoethanol and 250 mM imidazole, pH 8.0); the MBP-Miro1 protein was eluted using MBP elution buffer (20 mM Tris-HCl, 200 mM NaCl, 1 mM EDTA, 1 mM DTT (optional), 10 mM maltose, pH 7.5), and the GST-Kif5b and GST-Miro1 proteins were eluted using GST elution buffer (50 mM Tris-HCl, 250 mM NaCl, 50 mM GSH, pH 8.0).

For pull-down assays, purified bait proteins bound to beads were incubated with prey proteins for 2 h at 4 °C. After five washes, the bound complexes were eluted and analyzed by immunoblotting or Coomassie blue staining.

### In vitro phase separation and FRAP assays

The purified proteins were concentrated and buffer-exchanged into storage buffer (25 mM Tris-HCl (pH 8.0), 500 mM KCl, and 10% glycerol) using Amicon® Ultra filters (Millipore). After concentration, the protein was aliquoted and rapidly frozen in liquid nitrogen, followed by storage at −80 °C. The protein concentration was determined using a BCA protein assay kit (Mei5bio, MF071-01). For phase separation, proteins were diluted in low-salt buffer (25 mM Tris-HCl (pH 8.0) and 150 mM KCl) with or without PEG8000 and CaCl_2_ in 384-well plates. Droplets were visualized after the indicated time of incubation.

For the FRAP analysis of condensate assembly in vitro, particles were chosen for initial photobleaching with the laser corresponding to the fluorophore used to label the protein for 0.5 s with a laser power of 50%, and the fluorescence recovery was imaged at 2 s intervals for a total of ~1 min using a Zeiss LSM980 microscope.

### Quantification and statistical analysis

The asymmetry coefficient was analyzed as previously described^33^. For immunoblotting, immunofluorescence staining, and live-cell imaging, representative images from at least three independent biological experiments are shown. Statistical analyses were performed using GraphPad Prism. The data are presented as the means, with the error bars representing the indicated measures of variation. Statistical methods and sample sizes (*n*) for quantitative analyses are provided in the figure legends. Exact *P* values are indicated in the figures.

## Supplementary information

Supplementary Information

Supplementary Video S1

Supplementary Video S2
