# Supplementary material for: ER–mitochondria contacts orchestrate mitochondrial transport via C19orf12
Source: Cell Discov. 2026 Nov 10;12:80. doi: 10.1038/s41421-026-00934-0 (PMC13751628; doi:10.1038/s41421-026-00934-0)
Supplement: Supplementary file 1 [file 41421_2026_934_MOESM1_ESM.pdf]

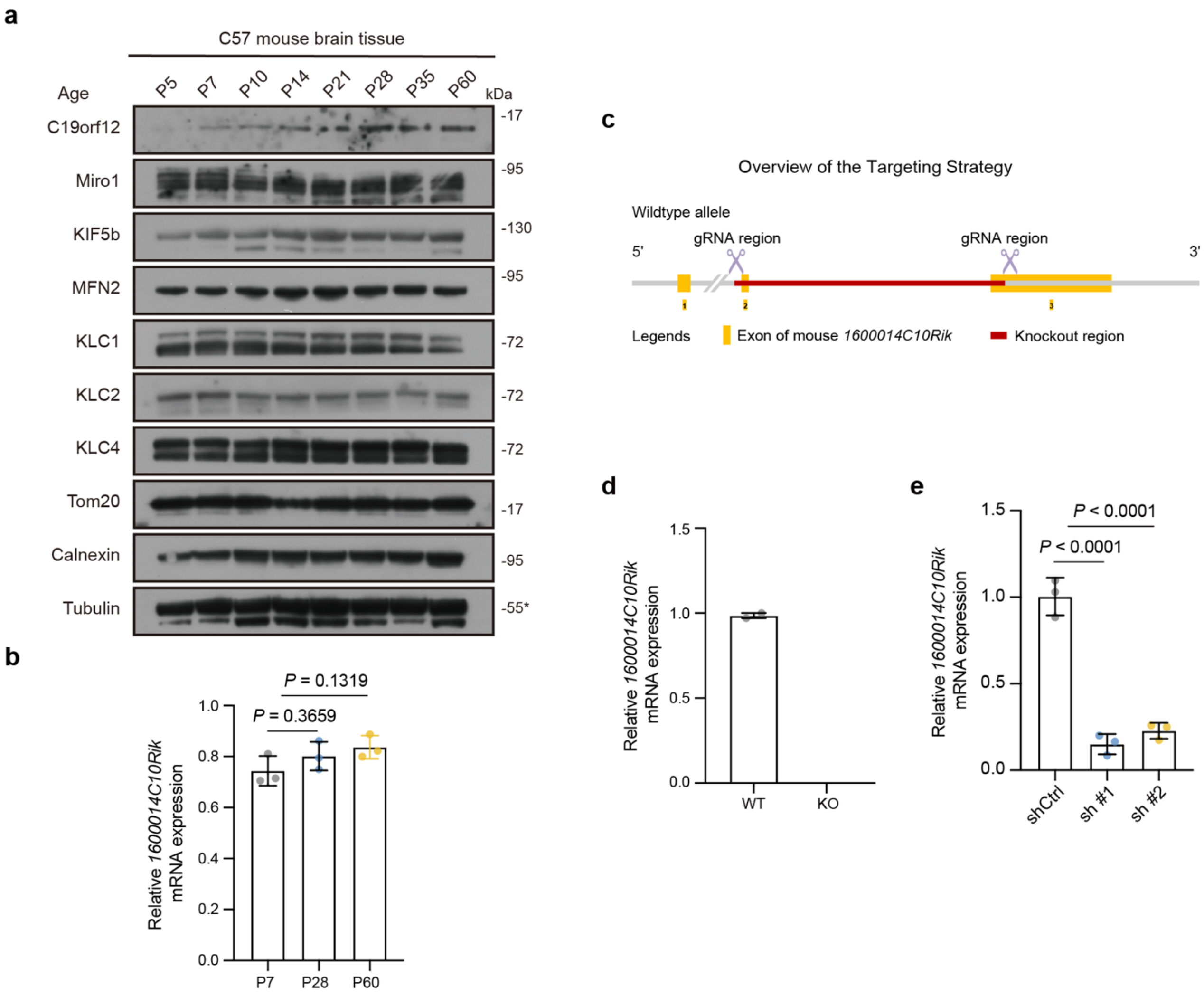
a
C57 mouse brain tissue
Age
P5
P7
P10
P14
P21
P28
P35
P60
kDa
C19orf12
Miro1
KIF5b
MFN2
KLC1
KLC2
KLC4
Tom20
Calnexin
Tubulin
-17
-95
-130
-95
-72
-72
-72
-17
-95
-55*
b
P = 0.1319
P = 0.3659
Relative 1600014C10Rik mRNA expression
P7
P28
P60
c
Overview of the Targeting Strategy
Wildtype allele
gRNA region
gRNA region
5'
3'
Legends
Exon of mouse 1600014C10Rik
Knockout region
d
Relative 1600014C10Rik mRNA expression
WT
KO
e
P < 0.0001
P < 0.0001
Relative 1600014C10Rik mRNA expression
shCtrl
sh #1
sh #2

Supplementary Fig. S1 | Characterization of C19orf12 expression and generation of knockout mice

a, Immunoblot analysis of brain lysates from wild-type C57 mice at the indicated developmental stages (P5 to P60), probed with antibodies against C19orf12 and the indicated mitochondrial transport-related proteins.

b, RT-qPCR analysis of *1600014C10Rik* mRNA levels in brain tissues from wild-type C57 mice at the indicated developmental stages (P7, P28, P60). $n = 3$ experiments. Data are shown as mean ± s.e.m. Each dot represents one biological replicate, the statistical significance was determined by Kruskal-Wallis test with multiple comparisons.

c, Schematic illustration of the CRISPR–Cas9 targeting strategy used to generate *1600014C10Rik* knockout mice. Two sgRNAs were designed to delete the exon of the mouse *1600014C10Rik* gene.

d, RT-qPCR validation of knockout efficiency. Analysis of *1600014C10Rik* mRNA levels confirms the complete absence of *1600014C10Rik* transcripts in knockout mice compared to wild-type littermates. n = 2 experiments. Data are presented as mean ± s.e.m.

e, RT–qPCR analysis of relative *1600014C10Rik* mRNA expression in primary mouse hippocampal neurons expressing shCtrl or either of two independent shRNAs targeting *1600014C10Rik* ($n = 3$ experiments). Data are shown as mean ± s.e.m. Each dot represents one biological replicate, and statistical significance was determined by one-way ANOVA with multiple comparisons.

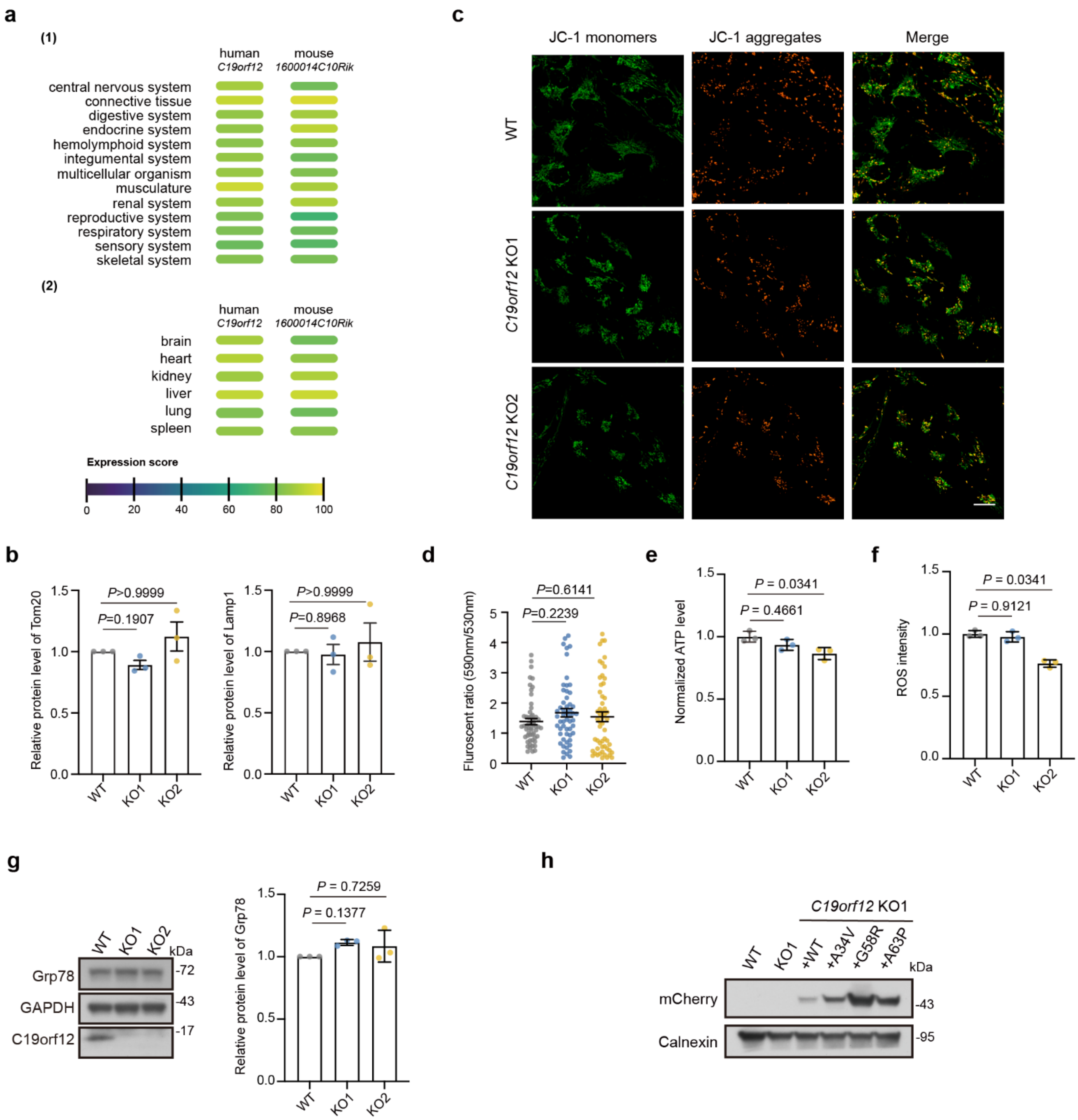
a
(1)
human C19orf12
mouse 1600014C10Rik
central nervous system
connective tissue
digestive system
endocrine system
hemolymphoid system
integumental system
multicellular organism
musculature
renal system
reproductive system
respiratory system
sensory system
skeletal system
(2)
brain
heart
kidney
liver
lung
spleen
Expression score
0 20 40 60 80 100
c
JC-1 monomers
JC-1 aggregates
Merge
WT
C19orf12 KO1
C19orf12 KO2
b
Relative protein level of Tom20
P>0.9999
P=0.1907
Relative protein level of Lamp1
P>0.9999
P=0.8968
WT KO1 KO2
d
Fluroscent ratio (590nm/530nm)
P=0.6141
P=0.2239
e
Normalized ATP level
P = 0.0341
P = 0.4661
f
ROS intensity
P = 0.0341
P = 0.9121
g
Grp78
GAPDH
C19orf12
kDa
-72
-43
-17
Relative protein level of Grp78
P = 0.7259
P = 0.1377
h
C19orf12 KO1
WT KO1 +WT +A34V +G58R +A63P
mCherry
Calnexin
-43
-95

Supplementary Fig. S2 | *C19orf12* is ubiquitously expressed, and its deficiency does not alter mitochondrial abundance or membrane potential

a, Hierarchical expression profile of human *C19orf12* and its mouse homolog (*1600014C10Rik*) across various anatomical systems **(1)** and major organs **(2)**, derived from the Bgee gene expression database. The color scale indicates the expression score (0–100).

b, Quantification of relative protein abundance of Tom20 (left) and LAMP1 (right) in WT and *C19orf12* KO U2OS cells shown in **Fig 3b**, normalized to WT levels. n = 3 experiments. Data are shown as mean ± s.e.m. Each dot represents one biological replicate, the statistical significance was determined by Kruskal-Wallis test with multiple comparisons.

c, d, JC-1 staining of U2OS WT and *C19orf12* KO cells showing mitochondrial membrane potential **(c)**, visualized as JC-1 monomers (green) and aggregates (orange), and quantification of the red/green fluorescence ratio **(d,** n = 55 per group**)**. Data are shown as mean ± s.e.m. Each dot represents one cell, and statistical significance was assessed by one-way ANOVA with multiple comparisons.

e, Quantification of relative total cellular ATP levels in WT and *C19orf12* KO1 and KO2 U2OS cells, normalized to WT levels. n = 3 experiments. Data are shown as mean ± s.e.m. Each dot represents one biological replicate, and statistical significance was determined by Kruskal–Wallis test with multiple comparisons.

f, Quantification of relative steady-state whole-cell ROS fluorescence intensity in WT and *C19orf12* KO1 and KO2 U2OS cells, normalized to WT levels. n = 3 experiments. Data are shown as mean ± s.e.m. Each dot represents one biological replicate, and statistical significance was determined by Kruskal–Wallis test with multiple comparisons.

g, Representative immunoblots (left) and quantification (right) of GRP78 protein abundance in WT and *C19orf12* KO1 and KO2 U2OS cells. GAPDH was used as the loading control, and C19orf12 immunoblotting confirmed gene knockout. GRP78 levels were normalized to GAPDH and are presented relative to WT levels. n = 3 experiments. Data are shown as mean ± s.e.m. Each dot represents one biological replicate, and statistical significance was determined by Kruskal–Wallis test with multiple comparisons.

h, Immunoblot analysis of mCherry-tagged C19orf12 constructs expressed in *C19orf12* KO1 U2OS cells. Calnexin was used as a loading control.

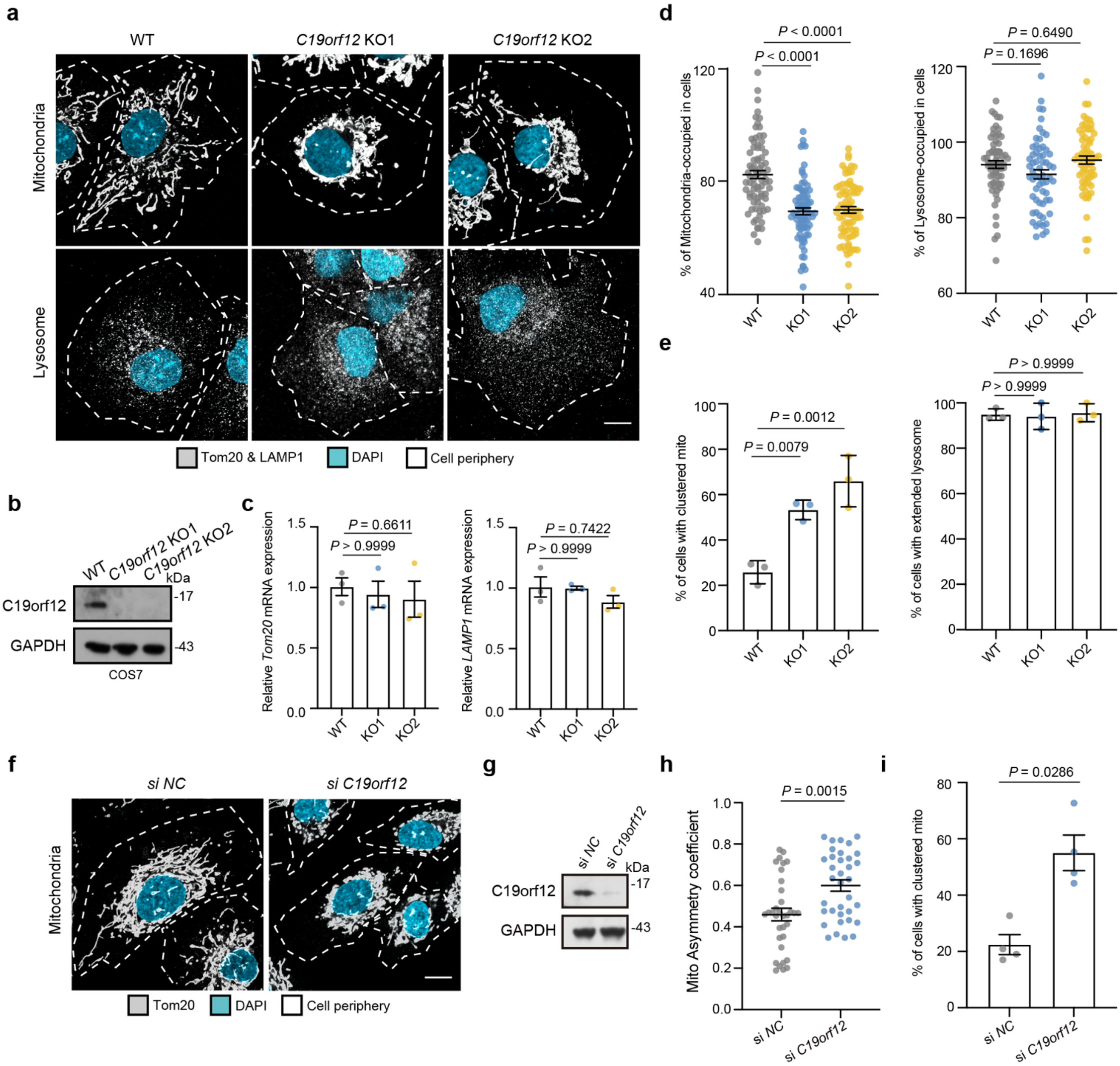

a
WT
C19orf12 KO1
C19orf12 KO2
Mitochondria
Lysosome
Tom20 & LAMP1
DAPI
Cell periphery
b
WT
C19orf12 KO1
C19orf12 KO2
kDa
C19orf12
-17
GAPDH
-43
COS7
c
P = 0.6611
P > 0.9999
Relative Tom20 mRNA expression
P = 0.7422
P > 0.9999
Relative LAMP1 mRNA expression
WT
KO1
KO2
d
P < 0.0001
P < 0.0001
% of Mitochondria-occupied in cells
P = 0.6490
P = 0.1696
% of Lysosome-occupied in cells
e
P = 0.0012
P = 0.0079
% of cells with clustered mito
P > 0.9999
P > 0.9999
% of cells with extended lysosome
f
si NC
si C19orf12
Mitochondria
Tom20
DAPI
Cell periphery
g
si NC
si C19orf12
kDa
C19orf12
-17
GAPDH
-43
h
P = 0.0015
Mito Asymmetry coefficient
i
P = 0.0286
% of cells with clustered mito

Supplementary Fig. S3 | C19orf12 deficiency induces conserved mitochondrial clustering in COS7 and A549 cells

a, Representative confocal images of COS7 WT and two independent *C19orf12* KO clones stained for mitochondria (Tom20, grey) and lysosomes (LAMP1, grey). Nuclei were labeled with DAPI (blue). Cell periphery is outlined by white dashed lines. Boxed regions indicate ROIs. Scale bars, 20 µm.

b, Immunoblot confirming loss of *C19orf12* in COS7 cells. GAPDH served as a loading control.

c, Quantitative PCR analysis showing unchanged mRNA level of Tom20 (left) and LAMP1 (right) in WT and *C19orf12* KO COS7 cells. $n = 3$ experiments. Data are shown as mean ± s.e.m. Each dot represents one biological replicate, the statistical significance was determined by Kruskal-Wallis test with multiple comparisons.

d, Percentage of cells occupied by mitochondria (left, $n = 77$ per group) or lysosomes (right, n = 67 per group) in WT and *C19orf12* KO COS7 cells. Data are shown as mean ± s.e.m. Each dot represents one cell and statistical significance was assessed by one-way ANOVA with multiple comparisons.

e, Quantification of the percentage of COS7 cells exhibiting perinuclear clustered mitochondria (left) or extended lysosomes (right) in WT and *C19orf12* KO cells. $n = 3$ experiments, with at least 150 cells counted per condition. Data are shown as mean ± s.e.m. Each dot represents one biological replicate, the statistical significance was determined by Kruskal-Wallis test with multiple comparisons.

f, Representative confocal images of mitochondria in A549 cells transfected with control siRNA (si NC) or siRNA targeting *C19orf12* (si *C19orf12*). Mitochondria were labeled with Tom20 (grey) and nuclei with DAPI (blue). Cell periphery is outlined by white dashed lines. Scale bars, 20 µm.

g, Immunoblot confirming *C19orf12* knockdown in A549 cells following siRNA treatment.

h, Quantification of mitochondrial asymmetry coefficients in si NC ($n = 34$) and si *C19orf12* ($n = 35$) A549 cells. Data are shown as mean ± s.e.m. Each dot represents one cell, the statistical significance was determined by Mann-Whitney test.

i, Quantification of the percentage of A549 cells exhibiting perinuclear mitochondrial clustering in si NC and si *C19orf12* cells. $n = 4$ experiments, with at least 150 cells counted per condition. Data are shown as mean ± s.e.m. Each dot represents one biological replicate, the statistical significance was determined by Mann-Whitney test.

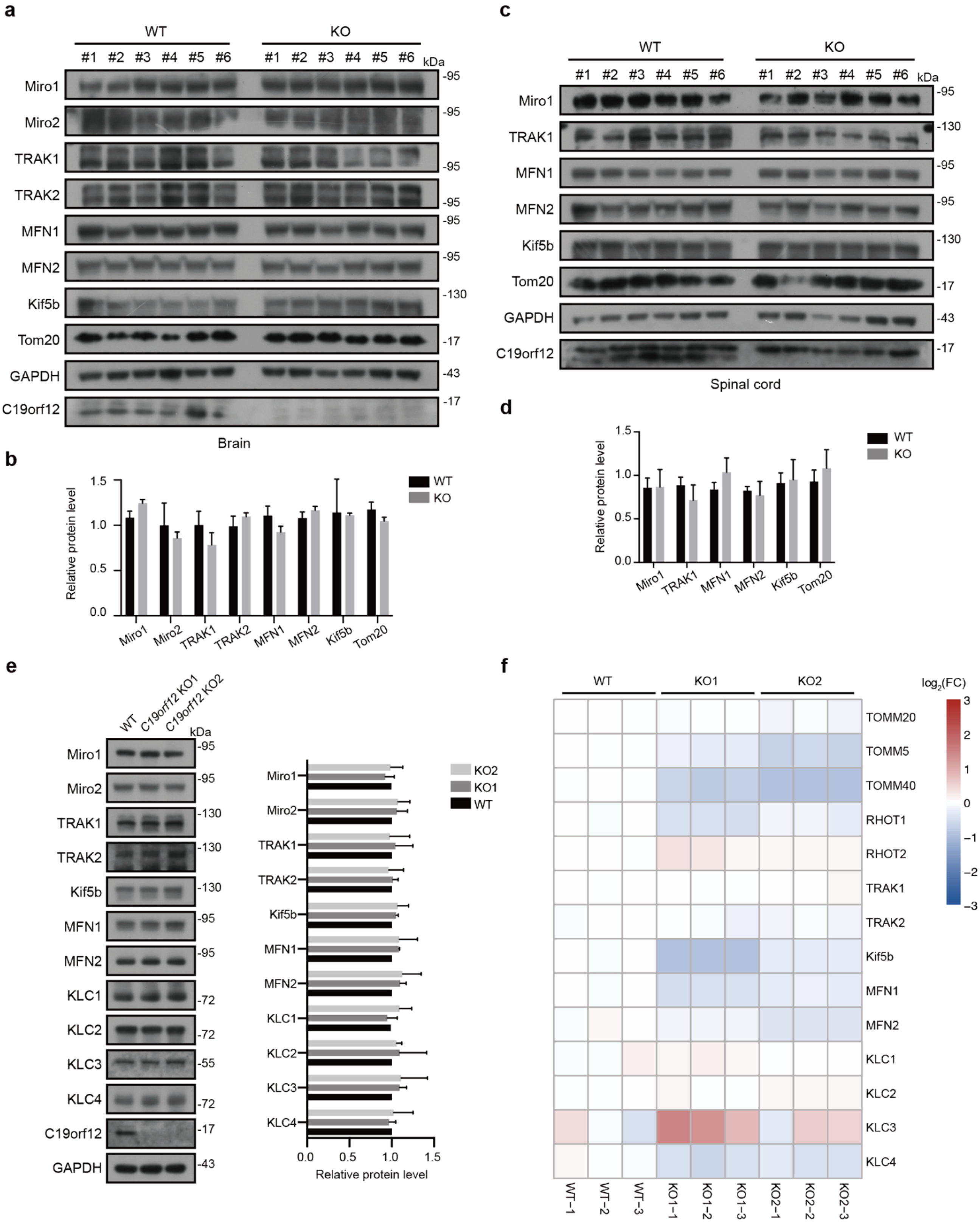

a
WT
KO
#1 #2 #3 #4 #5 #6
kDa
Miro1
Miro2
TRAK1
TRAK2
MFN1
MFN2
Kif5b
Tom20
GAPDH
C19orf12
-95
-130
-17
-43
Brain
b
Relative protein level
WT
KO
Miro1 Miro2 TRAK1 TRAK2 MFN1 MFN2 Kif5b Tom20
c
WT
KO
#1 #2 #3 #4 #5 #6
kDa
Miro1
TRAK1
MFN1
MFN2
Kif5b
Tom20
GAPDH
C19orf12
Spinal cord
d
Relative protein level
WT
KO
Miro1 TRAK1 MFN1 MFN2 Kif5b Tom20
e
WT
C19orf12 KO1
C19orf12 KO2
kDa
Miro1
Miro2
TRAK1
TRAK2
Kif5b
MFN1
MFN2
KLC1
KLC2
KLC3
KLC4
C19orf12
GAPDH
KO2
KO1
WT
Relative protein level
f
WT
KO1
KO2
log2(FC)
TOMM20
TOMM5
TOMM40
RHOT1
RHOT2
TRAK1
TRAK2
Kif5b
MFN1
MFN2
KLC1
KLC2
KLC3
KLC4
WT−1 WT−2 WT−3 KO1−1 KO1−2 KO1−3 KO2−1 KO2−2 KO2−3

Supplementary Fig. S4 | The abundance of transport machinery components remains unchanged in knockout mice and *C19orf12* KO cells

a, Immunoblot analysis of key mitochondrial transport proteins (Miro1, Miro2, TRAK1, TRAK2, Kif5b), fusion proteins (MFN1, MFN2), and the mitochondrial marker Tom20 in whole brain lysates from 12-month-old wild-type and knockout mice (n = 6 mice per group). GAPDH serves as a loading control.

b, Quantification of the relative protein levels shown in **(a)**. n = 4 experiments per group. Data represent mean ± s.e.m. The statistical significance was determined by multiple unpaired t test. Quantification (P = 0.1009, 0.6040, 0.3200, 0.4051, 0.1982, 0.3174, 0.9367, 0.2134 respectively) confirms that the abundance of each protein showed no significant difference across these independent replicates.

c, Immunoblot analysis of the same panel of transport and mitochondrial proteins in spinal cord lysates from wild-type and knockout mice (n = 6 mice per group). GAPDH serves as a loading control.

d, Quantification of the relative protein levels shown in **(c)**. n = 4 experiments per group. Data represent mean ± s.e.m. The statistical significance was determined by multiple unpaired t test. Quantification (P = 0.9703, 0.4236, 0.3244, 0.7703, 0.8854, 0.5632, respectively) confirms that the abundance of each protein showed no significant difference across these replicates.

e, Immunoblot analysis of mitochondrial transport associated proteins, including Miro1, Miro2, TRAK1, TRAK2, Kif5b, MFN1, MFN2 and KLC family members, in U2OS WT and *C19orf12* KO cells. GAPDH served as a loading control. Data represent mean ± s.e.m. from n = 3 independent experiments. Quantification confirms that the abundance of each protein showed no significant difference across three independent replicates

f, Heatmap showing relative expression levels of genes associated with mitochondrial transport across U2OS WT, *C19orf12* KO1 and *C19orf12* KO2 cells. Colors indicate the RNA-seq $\log_2$ fold changes relative to WT expression levels. FC, fold change.

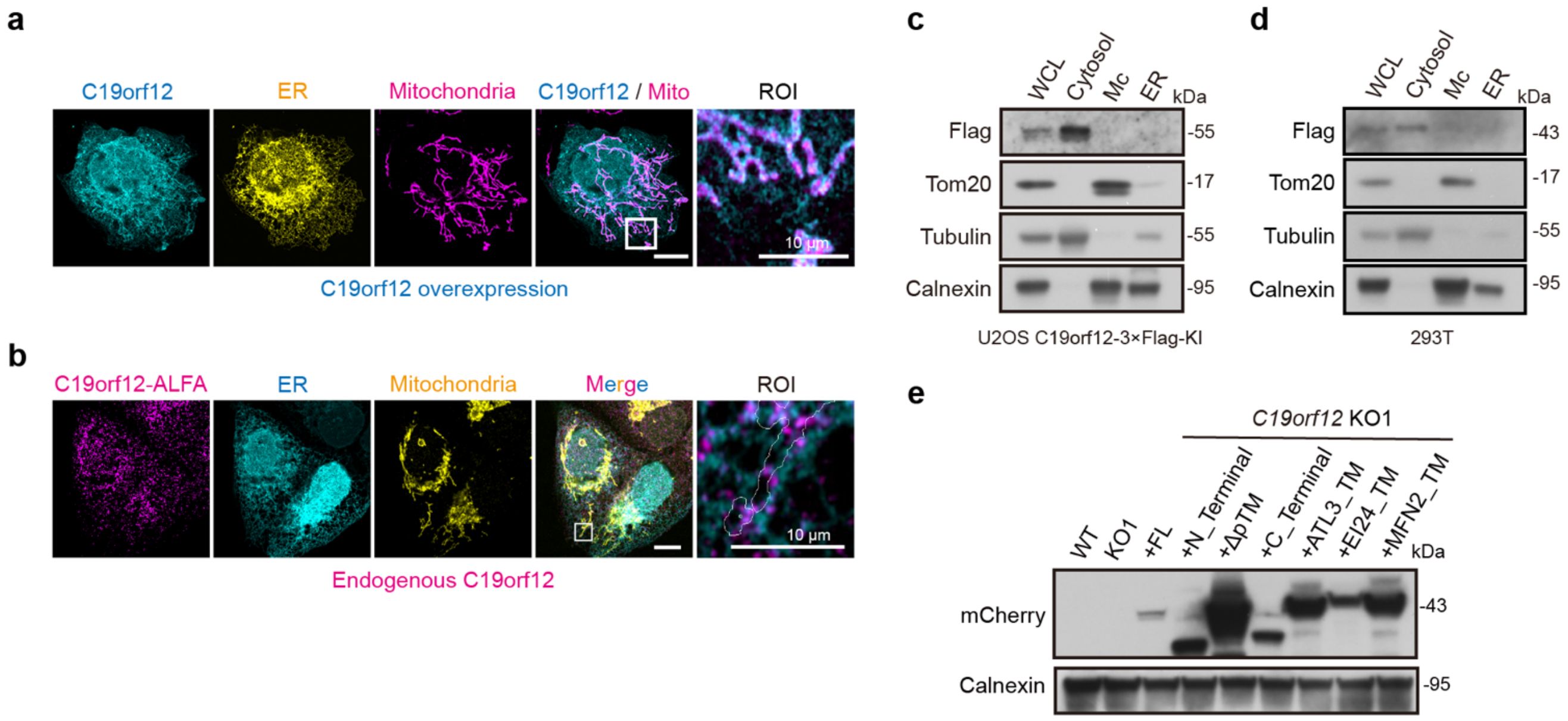

Supplementary Fig. S5 | Characterization of C19orf12 subcellular localization and expression of rescue constructs

a, Representative confocal images of U2OS cells co-transfected with C19orf12–mEmerald and the ER marker Sec61β (yellow), and stained for mitochondria (Tom20, magenta).

b, Representative confocal images of U2OS C19orf12–ALFA (magenta) knock-in (KI) cells transiently expressing the ER marker Sec61β (cyan) and stained for mitochondria (Tom20, yellow). The merged image shows C19orf12 localization relative to ER and mitochondria. Boxed regions indicate ROIs shown at higher magnification. Scale bars, 20 µm.

c-d, Subcellular fractionation of U2OS C19orf12–3×Flag KI cells **(c)** and 293T cells expressing C19orf12-Flag **(d)** followed by immunoblot analysis of whole-cell lysate (WCL), cytosol, crude mitochondria (Mc) and ER-enriched (ER) fractions. Tom20, Tubulin and Calnexin were used as mitochondrial, cytosolic and ER markers, respectively.

e, Immunoblot analysis confirming expression of mCherry-tagged full-length and mutant C19orf12 constructs in *C19orf12* KO U2OS cells. Calnexin served as a loading control.

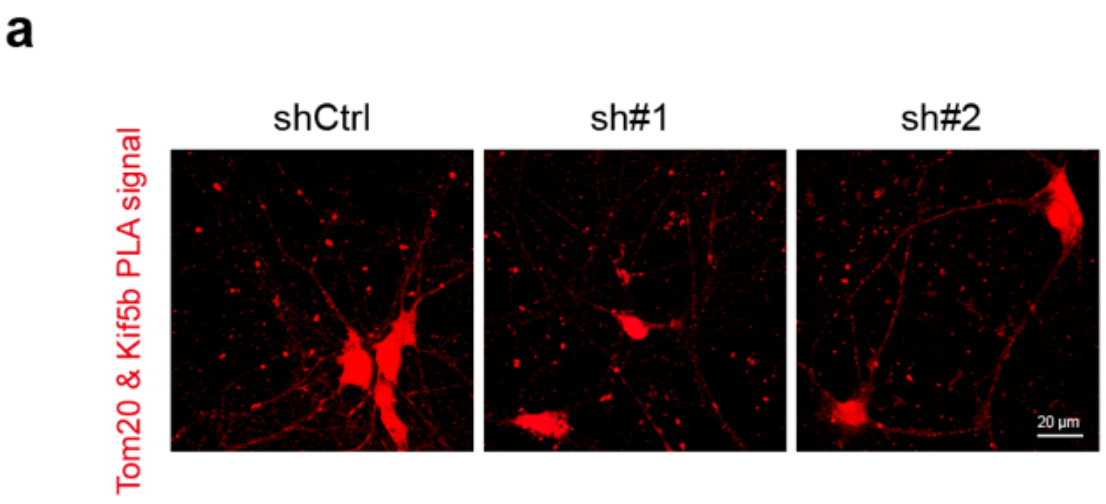

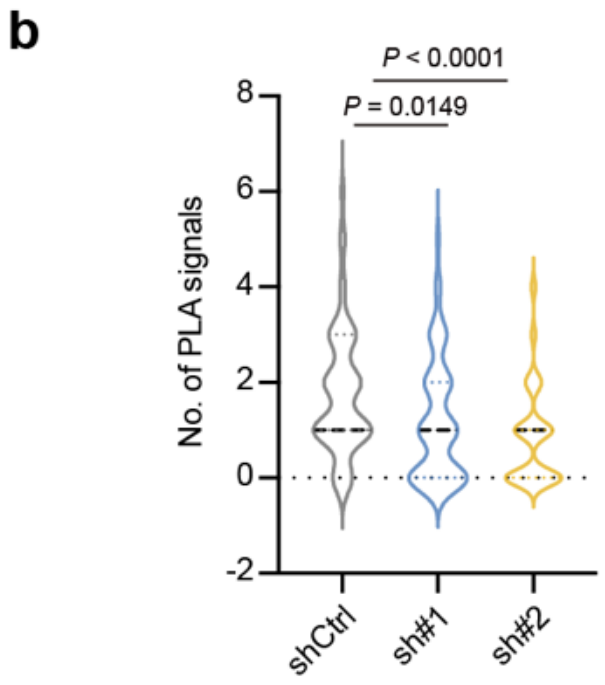

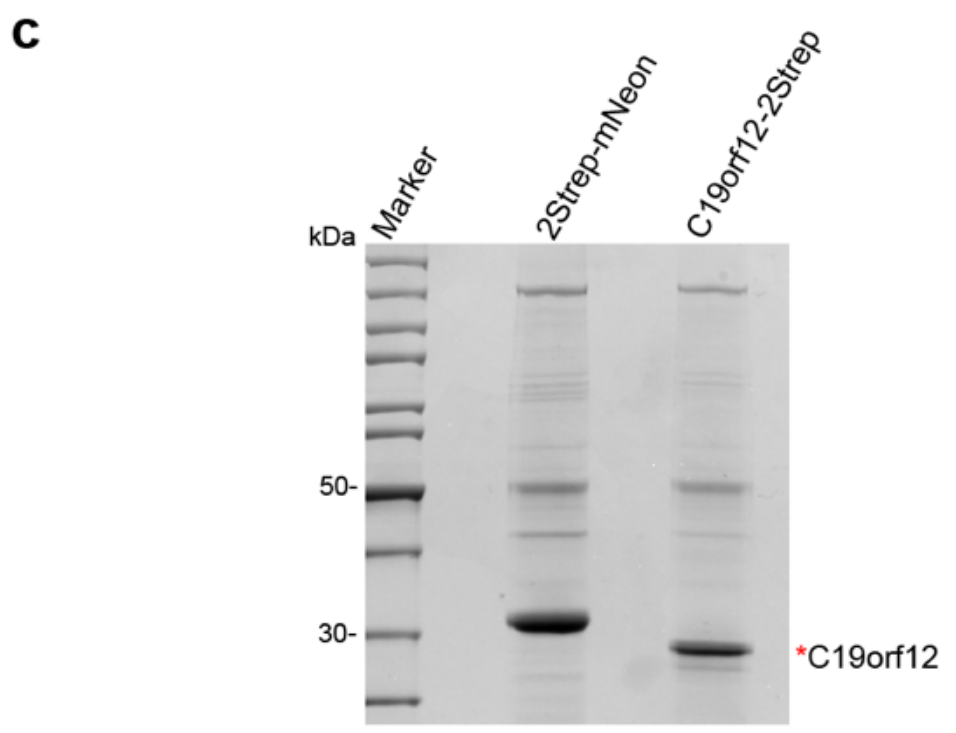

d

Protein name	PSM_Control	PSM_C19orf12
Kif5b	5	11
Miro1	1	4
Miro2	0	2

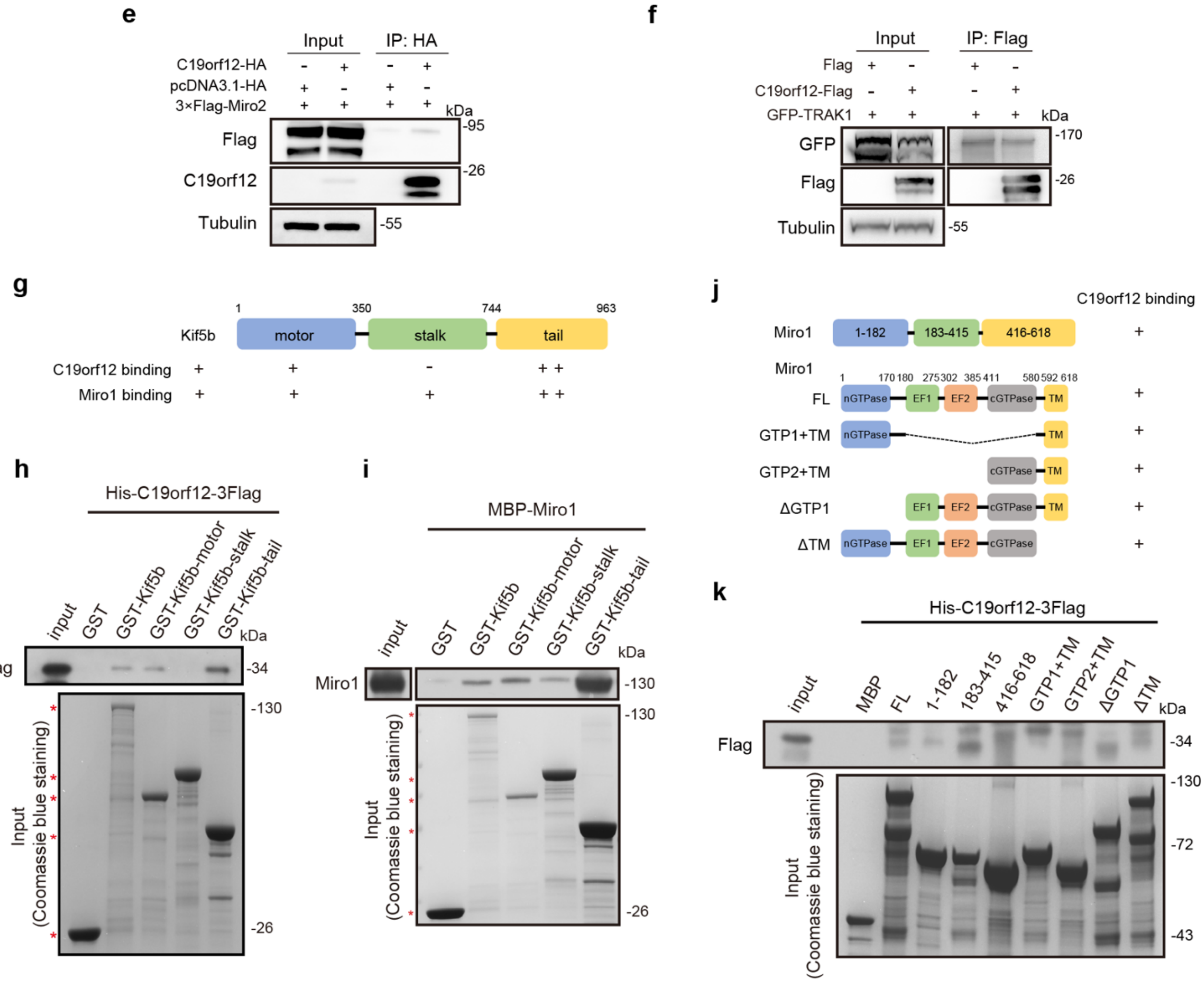

Supplementary Fig. S6 | Mapping of the interaction interfaces between C19orf12, Miro1 and Kif5b

a, Representative proximity ligation assay (PLA) images showing the proximity between endogenous Kif5b and the mitochondrial marker Tom20 in primary mouse hippocampal neurons transduced with control shRNA (shCtrl) or either of two independent shRNAs targeting *1600014C10Rik* (sh #1 and sh #2). PLA signals are shown in red. Scale bar, 20 μm.

b, Quantification of Kif5b–Tom20 PLA signals within the distal 30-μm regions of distal axons under the indicated conditions in **a** (n = 64, 62 and 60 axonal regions from left to right). Statistical significance was assessed by ordinary one-way ANOVA followed by multiple comparisons.

c, Coomassie blue staining of the purified Strep-tagged C19orf12 complex isolated from HEK293T cells used for AP-MS analysis. The band corresponding to C19orf12 is indicated.

d, List of peptide spectrum matches (PSMs) for key transport proteins identified in the AP-MS screen. Only Kif5b and Miros are shown.

e, Co-immunoprecipitation assays in 293T cells showing interaction between C19orf12 and Miro2.

f, Co-immunoprecipitation assays in 293T cells showing that there was no interaction between C19orf12 and TRAK1.

g, Schematic of Kif5b and its truncation constructs used in this study. Functional domains are indicated. C19orf12 and Miro1 binding abilities: +, positive; –, negative.

h, In vitro GST pull-down assays using purified His–C19orf12–3×Flag and GST-tagged full-length Kif5b or the indicated Kif5b truncation constructs. Input and bound fractions were analyzed by immunoblotting for Flag.

i, In vitro GST pull-down assays using MBP-tagged Miro1 and GST-tagged full-length Kif5b or the indicated Kif5b truncation constructs. Input and bound fractions were analyzed by immunoblotting for Miro1. Coomassie blue staining indicates the loading of purified proteins. In **h**, **i**, the position of each purified protein band is indicated by the asterisk adjacent to it.

j, Schematic of Miro1 Full-length (FL) and its truncation constructs used in this study. Functional domains are indicated. C19orf12 binding ability: +, positive.

k, MBP pull-down assays using purified His–C19orf12–3×Flag and the indicated Miro1 truncation constructs. Input and bound fractions were analyzed by immunoblotting for Flag. Coomassie blue staining indicates protein loading in **h-k**.

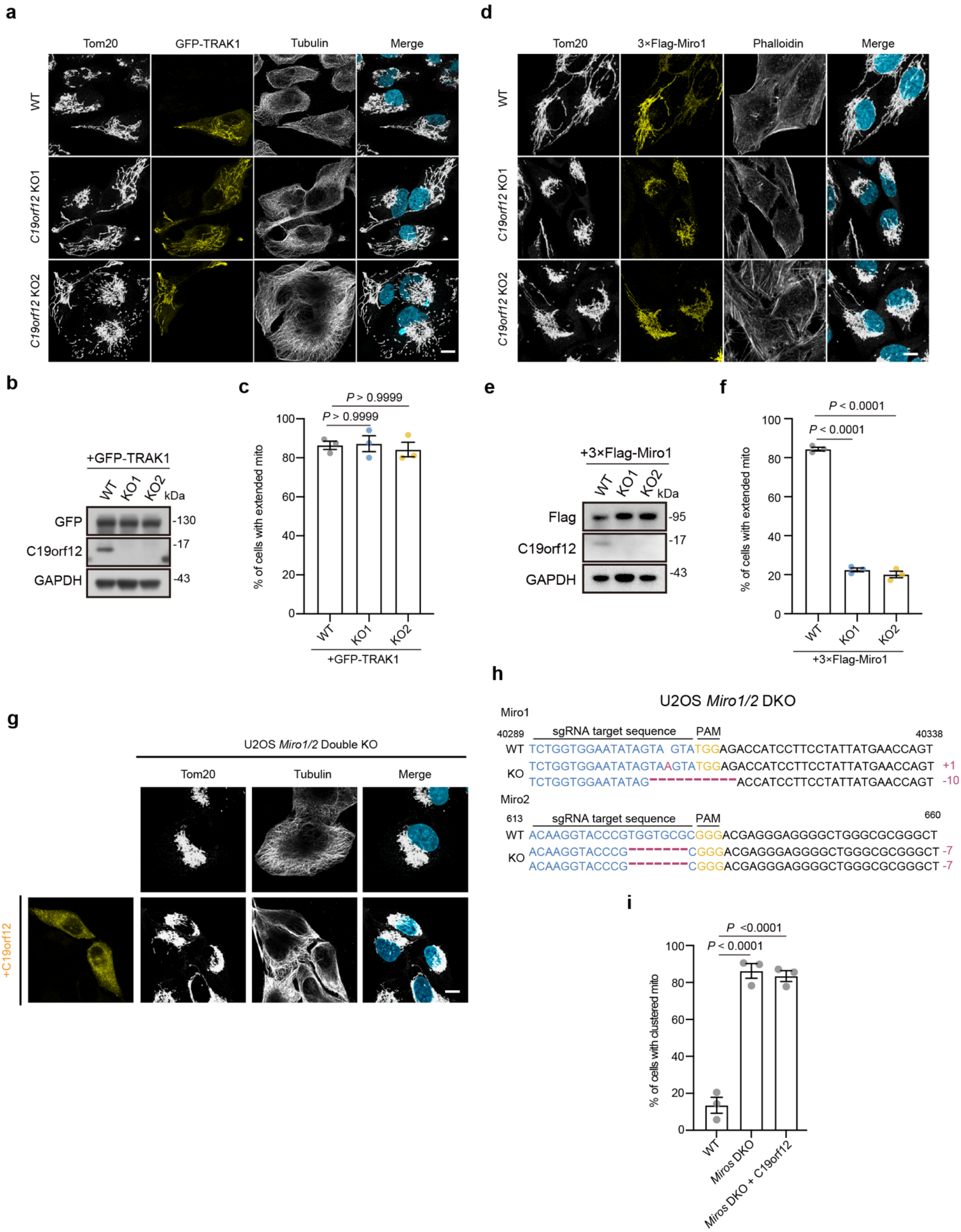

a
Tom20
GFP-TRAK1
Tubulin
Merge
WT
C19orf12 KO1
C19orf12 KO2
b
+GFP-TRAK1
WT KO1 KO2
kDa
GFP
-130
C19orf12
-17
GAPDH
-43
c
P > 0.9999
P > 0.9999
% of cells with extended mito
100
80
60
40
20
0
WT KO1 KO2
+GFP-TRAK1
d
Tom20
3×Flag-Miro1
Phalloidin
Merge
WT
C19orf12 KO1
C19orf12 KO2
e
+3×Flag-Miro1
WT KO1 KO2
kDa
Flag
-95
C19orf12
-17
GAPDH
-43
f
P < 0.0001
P < 0.0001
% of cells with extended mito
100
80
60
40
20
0
WT KO1 KO2
+3×Flag-Miro1
g
U2OS Miro1/2 Double KO
Tom20
Tubulin
Merge
+C19orf12
h
U2OS Miro1/2 DKO
Miro1
40289
sgRNA target sequence
PAM
40338
WT TCTGGTGGAATATAGTA GTATGGAGACCATCCTTCCTATTATGAACCAGT
KO TCTGGTGGAATATAGTAAGTATGGAGACCATCCTTCCTATTATGAACCAGT +1
TCTGGTGGAATATAG----------ACCATCCTTCCTATTATGAACCAGT -10
Miro2
613
sgRNA target sequence
PAM
660
WT ACAAGGTACCCGTGGTGCGCGGGACGAGGGAGGGGCTGGGCGCGGGCT
KO ACAAGGTACCCG-------CGGGACGAGGGAGGGGCTGGGCGCGGGCT -7
ACAAGGTACCCG-------CGGGACGAGGGAGGGGCTGGGCGCGGGCT -7
i
P <0.0001
P < 0.0001
% of cells with clustered mito
100
80
60
40
20
0
WT
Miros DKO
Miros DKO + C19orf12

Supplementary Fig. S7 | C19orf12-dependent mitochondrial redistribution is independent of TRAK1

a, d, Representative confocal images of U2OS WT and *C19orf12* KO cells transiently expressing GFP–TRAK1 **(a)** and 3×Flag-Miro1 **(d)** and stained for mitochondria (Tom20) and Tubulin **(a)** or Phalloidin **(d)**. Scale bars, 20 µm.

b, e Immunoblot analysis confirming expression of GFP-TRAK1 **(b)** and 3×Flag-Miro1 **(e)** in WT and *C19orf12* KO U2OS cells. GAPDH served as a loading control.

c, f, Quantification of the percentage of cells exhibiting perinuclear clustering of mitochondria under the indicated conditions in **a** and **d**. n = 3 experiments, with at least 90 cells counted per condition. Each dot represents one biological replicate. Data are shown as mean ± s.e.m.; the statistical significance was determined by Kruskal-Wallis test with multiple comparisons.

g, Representative confocal images of mitochondria (Tom20) and tubulin in U2OS cells with double knockout of *Miro1* and *Miro2*, with or without re-expression of C19orf12. Scale bars, 20 µm.

h, Genome sequencing validation of the sgRNA-induced indels in the *Miro1* and *Miro2* loci of the DKO cell line.

i, Quantification of the percentage of cells exhibiting perinuclear clustering of mitochondria under the indicated conditions in **g**. n = 3 experiments, with at least 90 cells counted per condition. Each dot represents one biological replicate. Data are shown as mean ± s.e.m.; the statistical significance was determined by Kruskal-Wallis test with multiple comparisons.

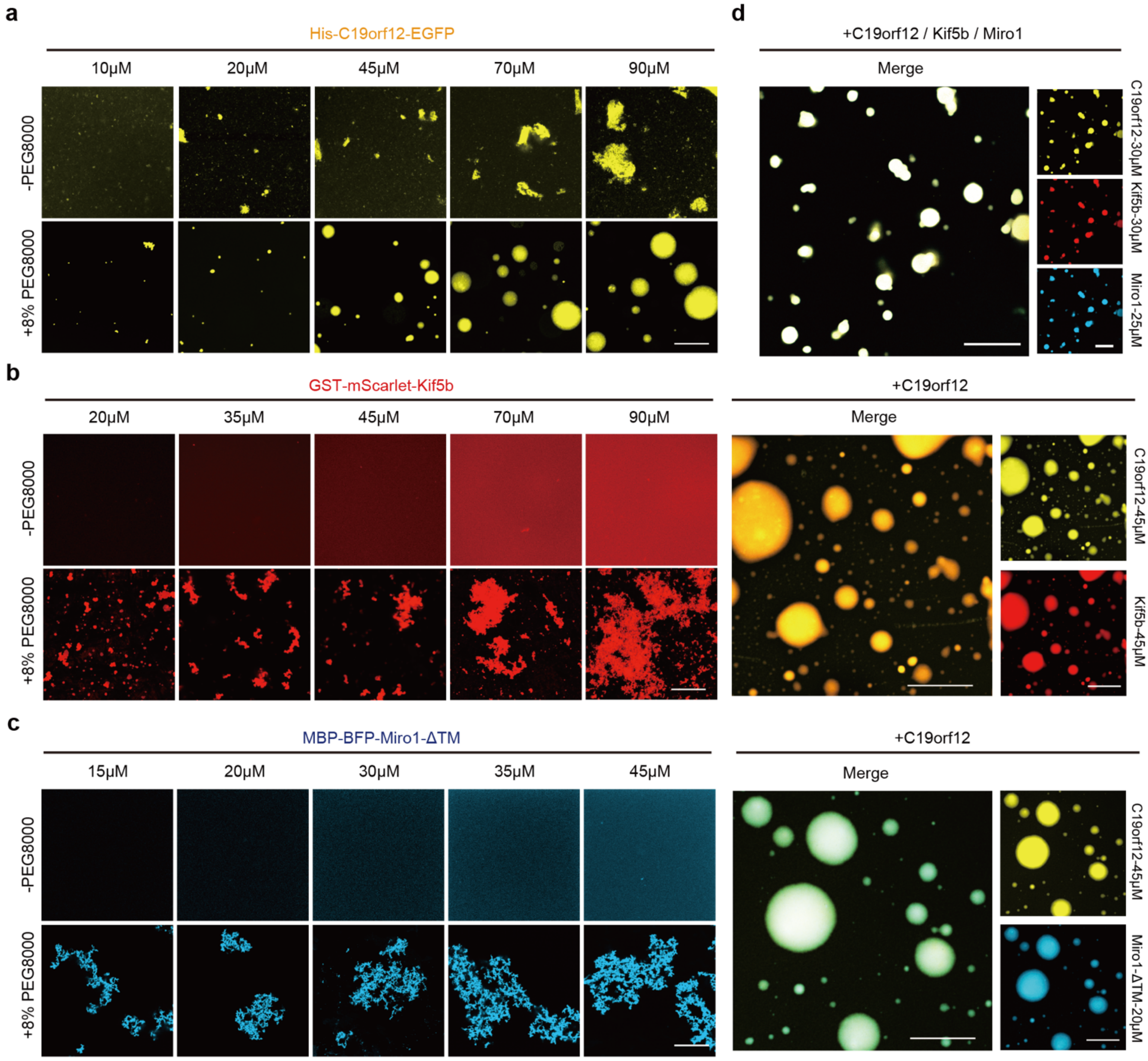

a
His-C19orf12-EGFP
10μM
20μM
45μM
70μM
90μM
-PEG8000
+8% PEG8000
b
GST-mScarlet-Kif5b
20μM
35μM
45μM
70μM
90μM
-PEG8000
+8% PEG8000
c
MBP-BFP-Miro1-ΔTM
15μM
20μM
30μM
35μM
45μM
-PEG8000
+8% PEG8000
d
+C19orf12 / Kif5b / Miro1
Merge
C19orf12-30μM
Kif5b-30μM
Miro1-25μM
+C19orf12
Merge
C19orf12-45μM
Kif5b-45μM
+C19orf12
Merge
C19orf12-45μM
Miro1-ΔTM-20μM

Supplementary Fig. S8 | C19orf12 undergoes phase separation and co-condenses with mitochondrial transport proteins

a, Representative fluorescence images of purified His–C19orf12–EGFP at the indicated protein concentrations in buffer containing 25 mM Tris-HCl, 150 mM KCl, in the absence or presence of 8% PEG8000. Spherical condensates form under crowding conditions in a concentration-dependent manner. Scale bars, 20 μm.

b, Representative fluorescence images of purified GST–mScarlet–Kif5b at the indicated protein concentrations under the same buffer conditions as in **a**, in the absence or presence of 8% PEG8000. Scale bars, 20 μm.

c, Representative fluorescence images of purified MBP–BFP–Miro1ΔTM at the indicated protein concentrations under the same buffer conditions as in **a**, in the absence or presence of 8% PEG8000. Scale bars, 20 μm.

d, Representative fluorescence images showing co-condensation of His–C19orf12–EGFP with GST–mScarlet–Kif5b and MBP–BFP–Miro1–ΔTM in vitro under crowding conditions. Merged and single-channel images are shown, indicating co-enrichment of C19orf12, Kif5b and Miro1 within the same condensates. Scale bars, 20 μm.

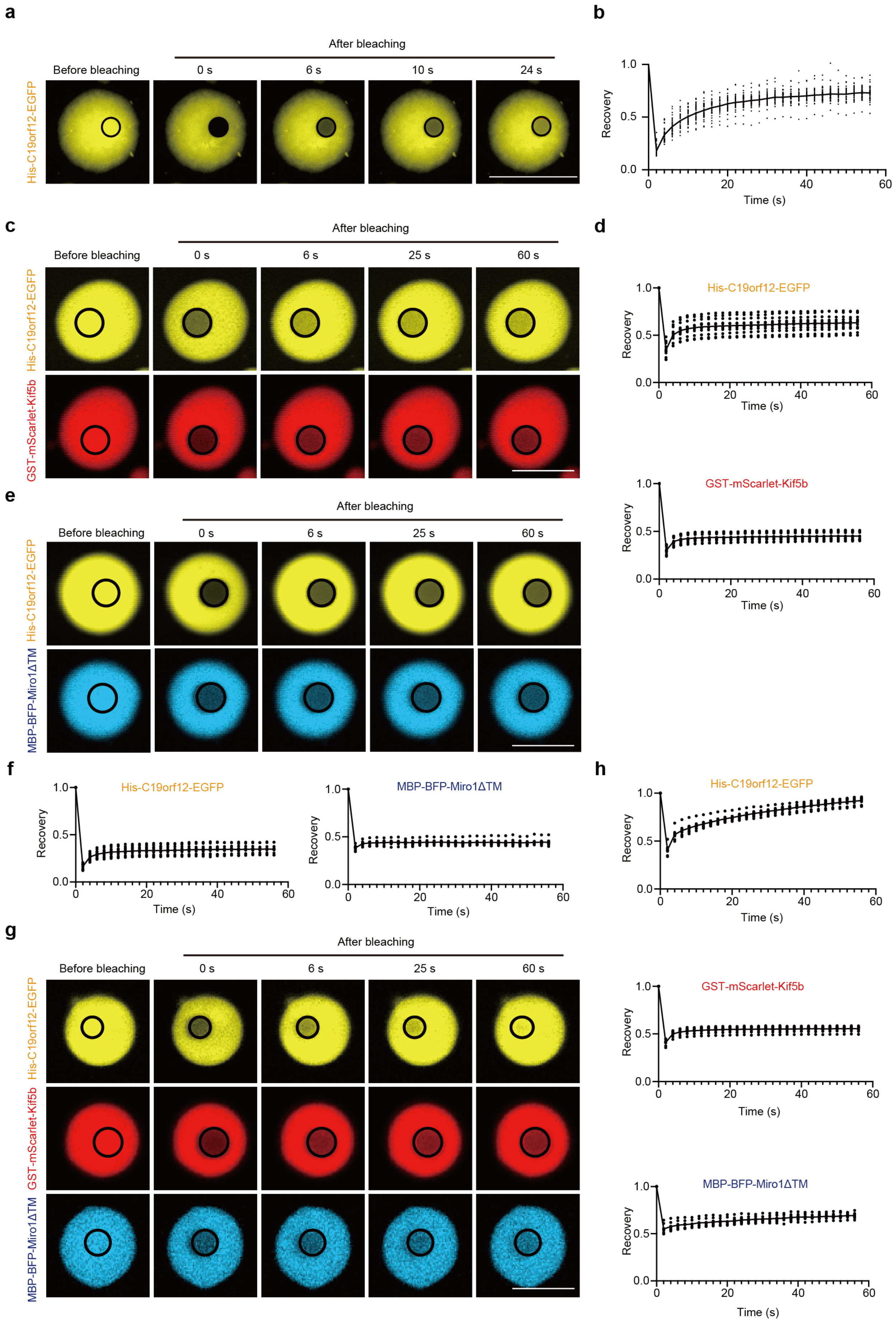

a
After bleaching
Before bleaching
0 s
6 s
10 s
24 s
His-C19orf12-EGFP
b
Recovery
Time (s)
c
After bleaching
Before bleaching
0 s
6 s
25 s
60 s
His-C19orf12-EGFP
GST-mScarlet-Kif5b
d
His-C19orf12-EGFP
GST-mScarlet-Kif5b
Recovery
Time (s)
e
After bleaching
Before bleaching
0 s
6 s
25 s
60 s
His-C19orf12-EGFP
MBP-BFP-Miro1ΔTM
f
His-C19orf12-EGFP
MBP-BFP-Miro1ΔTM
Recovery
Time (s)
g
After bleaching
Before bleaching
0 s
6 s
25 s
60 s
His-C19orf12-EGFP
GST-mScarlet-Kif5b
MBP-BFP-Miro1ΔTM
h
His-C19orf12-EGFP
GST-mScarlet-Kif5b
MBP-BFP-Miro1ΔTM
Recovery
Time (s)

Supplementary Fig. S9 | Dynamic properties of C19orf12-containing condensates revealed by FRAP

a, Representative fluorescence recovery after photobleaching (FRAP) images of His–C19orf12–EGFP condensates formed in vitro. Images are shown before bleaching and at the indicated time points after bleaching. The bleached region is indicated by a circle. Scale bars, 5 µm.

b, Quantification of fluorescence recovery of His–C19orf12–EGFP condensates shown in **a** (n = 38). Dots represent individual replicates, with lines connecting the mean values over time.

c, e, Representative FRAP images of condensates containing His–C19orf12–EGFP together with GST–mScarlet–Kif5b **(c)** or MBP–BFP–Miro1ΔTM **(e)** formed in vitro. Images are shown before bleaching and at the indicated time points after bleaching. The bleached regions are indicated by circles. Scale bars, 5 µm.

d, f, Quantification of fluorescence recovery for His–C19orf12–EGFP and GST–mScarlet–Kif5b in condensates shown in **c (d,** n = 10**)**, and for His–C19orf12–EGFP and MBP–BFP–Miro1ΔTM in condensates shown in **e (f,** n = 10**)**. Dots represent individual replicates, with lines connecting the mean values over time.

g, Representative FRAP images of condensates containing His–C19orf12–EGFP, GST–mScarlet–Kif5b and MBP–BFP–Miro1ΔTM formed in vitro. Images are shown before bleaching and at the indicated time points after bleaching. Scale bars, 5 µm.

h, Quantification of fluorescence recovery of His–C19orf12–EGFP (top), GST–mScarlet–Kif5b (middle) and MBP–BFP–Miro1ΔTM (bottom) in condensates shown in **g** (**h**, n = 10). Dots represent individual replicates, with lines connecting the mean values over time.

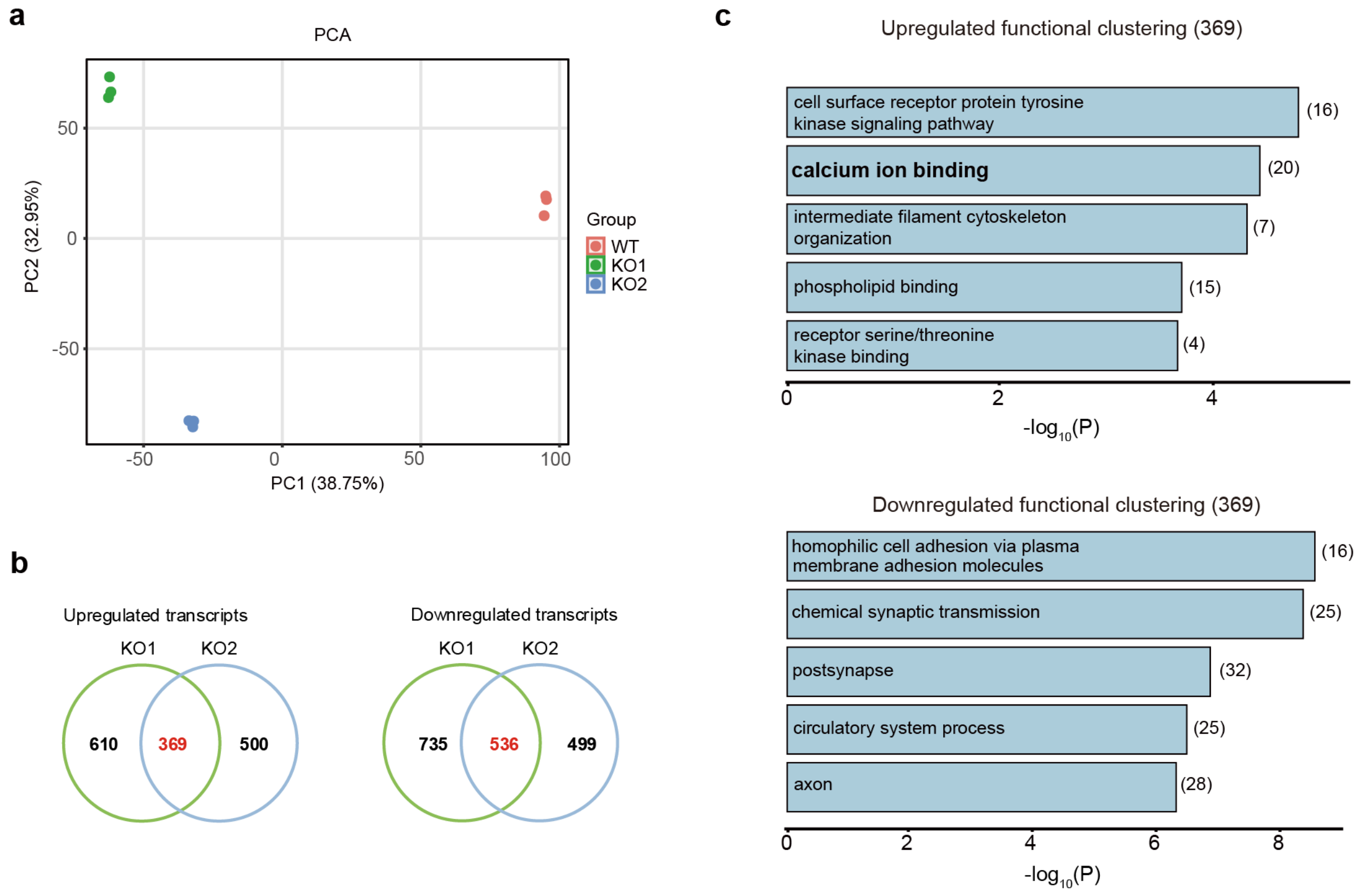

Supplementary Fig. S10 | Transcriptional analysis of U2OS WT and *C19orf12* KO cells

a, Principal component analysis (PCA) of RNA-seq data from U2OS WT and two independent *C19orf12* KO cells. PC, principal component.

b, Venn diagrams showing the overlap of upregulated (left) and downregulated (right) differentially expressed genes (DEGs) identified in U2OS *C19orf12* KO1 and *C19orf12* KO2 cells.

c, Functional enrichment analysis of the 369 commonly upregulated DEGs (top) and 536 commonly downregulated DEGs (bottom) identified in **b**. Bars represent enriched terms ranked by statistical significance, plotted as $-\log_{10}(P)$. Numbers in parentheses indicate the number of genes associated with each term. Enrichment analysis was performed using Metascape.

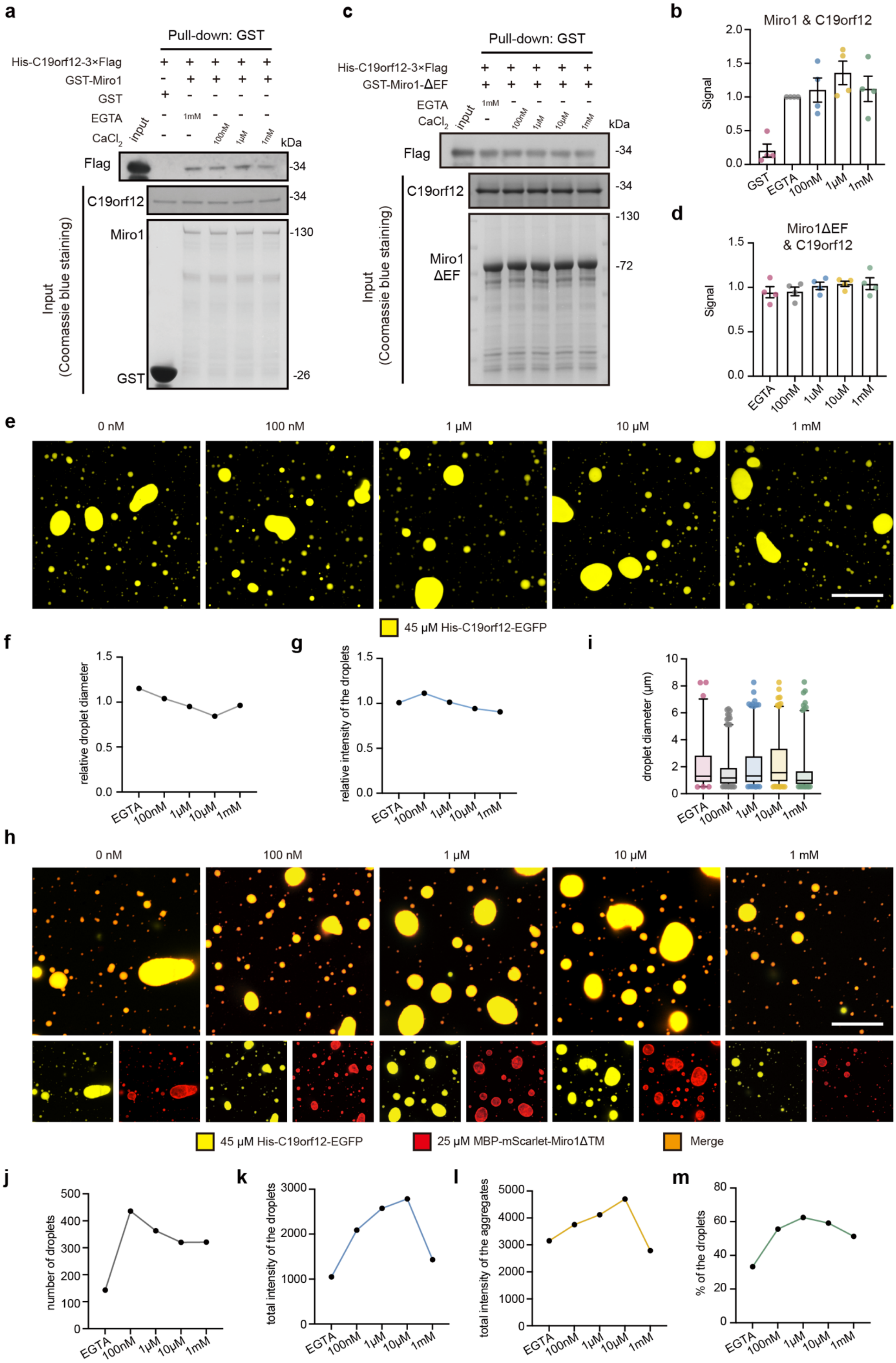

a
Pull-down: GST
His-C19orf12-3×Flag
GST-Miro1
GST
EGTA
CaCl2
input
kDa
Flag
C19orf12
Miro1
GST
Input (Coomassie blue staining)
c
His-C19orf12-3×Flag
GST-Miro1-ΔEF
Miro1 ΔEF
b
Miro1 & C19orf12
Signal
d
Miro1ΔEF & C19orf12
e
0 nM
100 nM
1 μM
10 μM
1 mM
45 μM His-C19orf12-EGFP
f
relative droplet diameter
g
relative intensity of the droplets
i
droplet diameter (μm)
h
25 μM MBP-mScarlet-Miro1ΔTM
Merge
j
number of droplets
k
total intensity of the droplets
l
total intensity of the aggregates
m
% of the droplets
EGTA
100nM
1μM
10μM
1mM

Supplementary Fig. S11 | Calcium modulates C19orf12-Miro1 association and co-condensation in vitro

a, c, In vitro GST pull-down assays examining the interaction between purified His–C19orf12–3×Flag and GST–Miro1 **(a)** or GST-Miro1-ΔEF-hand **(c)**in the presence of the indicated concentrations of $CaCl_2$ or EGTA. Input and bound fractions were analyzed by immunoblotting for Flag. Coomassie blue staining indicates protein loading.

b, d, Quantification of C19orf12–Miro1 **(b,** *n* = 4 experiments**)** and C19orf12-Miro1-ΔEF-hand **(d,** *n* = 3 experiments**)** interaction signals from pull-down assays shown in **a, c**. Data are shown as mean ± s.e.m. from independent experiments.

e, Representative fluorescence images of in vitro droplets formed by His–C19orf12–EGFP alone in the presence of indicated free Ca^{2+} concentrations ranging from 0 (EGTA) to 1 mM. Scale bars, 20 μm.

f, g, Quantification of the C19orf12 phase separation behavior shown in **e**. Analyses include the relative droplet diameter **(f)** and relative mean fluorescence intensity of the droplets **(g)**. Sample sizes (number of droplets analyzed) for EGTA, 100 nM, 1 μM, 10 μM, and 1 mM conditions are *n* = 4472, 6939, 5514, 5399, 6271, respectively.

h, Representative fluorescence images of in vitro condensates formed by His–C19orf12–EGFP and MBP–mScarlet–Miro1ΔTM at the indicated Ca^{2+} concentrations. Merged and single-channel images are shown. Scale bars, 20 μm.

i-m, Quantification of the co-condensation behavior shown in **h**. Analyses include the distribution of droplet diameters **(i)**, total number of droplets **(j)**, total fluorescence intensity within C19orf12 droplets **(k)**, total intensity of all C19orf12 aggregates **(l)**, and the proportion of C19orf12 protein in the condensed phase **(m)**. In **i**, data are presented as box-and-whisker plots: the center line represents the median, the box limits represent the 2.5 and 97.5 percentiles. Sample sizes (number of droplets analyzed) for EGTA, 100 nM, 1 μM, 10 μM, and 1 mM conditions are *n* = 143, 436, 363, 320 and 321, respectively.

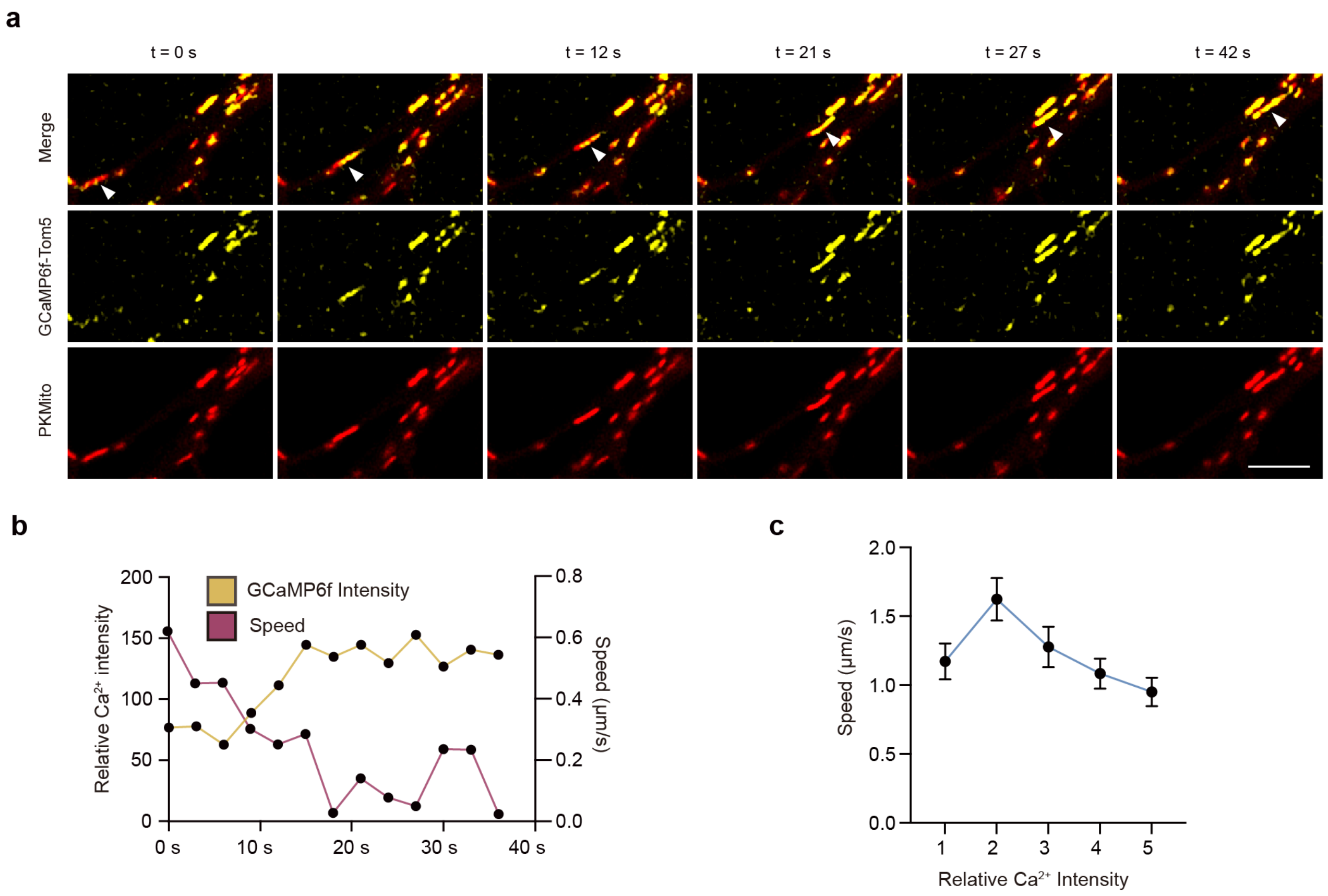
a
t = 0 s
t = 12 s
t = 21 s
t = 27 s
t = 42 s
Merge
GCaMP6f-Tom5
PKMito
b
GCaMP6f Intensity
Speed
Relative Ca2+ intensity
Speed (μm/s)
200
150
100
50
0
0.8
0.6
0.4
0.2
0.0
0 s
10 s
20 s
30 s
40 s
c
Speed (μm/s)
2.0
1.5
1.0
0.5
0.0
1
2
3
4
5
Relative Ca2+ Intensity

Supplementary Fig. S12 | Mitochondrial-surface Ca^{2+} signals exhibit a biphasic relationship with mitochondrial motility

a, Representative time-lapse images of axonal mitochondria labeled with PKMito (red) and Ca^{2+} signals reported by transiently expressed GCaMP6f–Tom5 (yellow) in living cells. Arrowheads indicate a representative mitochondrion exhibiting a characteristic transient Ca^{2+} signal over time. Scale bars, 20 μm.

b, Representative traces showing temporal changes in relative mitochondrial Ca^{2+} intensity (GCaMP6f–Tom5) and mitochondrial movement speed from the individual mitochondrion indicated by arrowheads in **a**.

c, Quantification of mitochondrial movement speed grouped by relative surface calcium intensity levels. All relative Ca^{2+}-intensity measurements were ranked from lowest to highest and divided into five equally sized groups (Bins 1–5), with higher bin numbers representing stronger relative Ca^{2+} signals. $n = 52$ per group. Data are shown as mean ± s.e.m. in every group.

Supplementary Video S1 | Dynamic movement of C19orf12 along the ER network

Single-molecule tracking of C19orf12 in U2OS *C19orf12*-Halo KI cells transiently expressing Sec61β (yellow). Representative trajectories show mobile C19orf12 molecules moving along the ER network. The video corresponds to the Fig. 4d.

Supplementary Video S2 | Dynamic movement of C19orf12 relative to mitochondria

Single-molecule tracking of C19orf12 in U2OS *C19orf12*-Halo KI cells labeled with the mitochondrial dye PKMito (cyan). Representative trajectories show the dynamic movement of C19orf12 relative to mitochondria. The video corresponds to the Fig. 4d.
